# Synergism between soluble guanylate cyclase signaling and neuropeptides extends lifespan in the nematode *Caenorhabditis elegans*

**DOI:** 10.1111/acel.12569

**Published:** 2017-01-04

**Authors:** Rachel Abergel, Leonid Livshits, Maayan Shaked, Arijit Kumar Chatterjee, Einav Gross

**Affiliations:** ^1^ Department of Biochemistry and Molecular BiologyIMRICFaculty of MedicineThe Hebrew University of JerusalemEin Kerem. P.O. Box 12271Jerusalem9112102Israel

**Keywords:** *Caenorhabditis elegans*, lifespan, NPR‐1, oxygen sensing, reactive oxygen species, soluble guanylate cyclase

## Abstract

Oxygen (O_2_) homeostasis is important for all aerobic animals. However, the manner by which O_2_ sensing and homeostasis contribute to lifespan regulation is poorly understood. Here, we use the nematode *Caenorhabditis elegans* to address this question. We demonstrate that a loss‐of‐function mutation in the neuropeptide receptor gene *npr‐1* and a deletion mutation in the atypical soluble guanylate cyclase *gcy‐35* O_2_ sensor interact synergistically to extend worm lifespan. The function of *npr‐1* and *gcy‐35* in the O_2_‐sensing neurons AQR, PQR, and URX shortens the lifespan of the worm. By contrast, the activity of the atypical soluble guanylate cyclase O_2_ sensor *gcy‐33* in these neurons is crucial for lifespan extension. In addition to AQR, PQR, and URX, we show that the O_2_‐sensing neuron BAG and the interneuron RIA are also important for the lifespan lengthening. Neuropeptide processing by the proprotein convertase EGL‐3 is essential for lifespan extension, suggesting that the synergistic effect of joint loss of function of *gcy‐35* and *npr‐1* is mediated through neuropeptide signal transduction. The extended lifespan is regulated by hypoxia and insulin signaling pathways, mediated by the transcription factors HIF‐1 and DAF‐16. Moreover, reactive oxygen species (ROS) appear to play an important function in lifespan lengthening. As HIF‐1 and DAF‐16 activities are modulated by ROS, we speculate that joint loss of function of *gcy‐35* and *npr‐1* extends lifespan through ROS signaling.

## Introduction

To survive, animals must sense key environmental factors, including temperature, food, and oxygen (O_2_). Sensory information then regulates homeostatic responses to maintain physiological parameters within a narrow range (Zimmer *et al*., 2009). O_2_ and reactive oxygen species (ROS) are important for animal viability but may be toxic when misregulated (Halliwell & Gutteridge, 2006).

The *Caenorhabditis elegans* (*C. elegans*) nematode uses atypical soluble guanylate cyclases (sGCs) to sense changes in O_2_ levels. The sGCs GCY‐35 and GCY‐36 are expressed in the O_2_‐sensing neurons AQR, PQR, and URX and appear to be activated by hyperoxia ([O2]>12%) (Gray *et al*., 2004; Abergel *et al*., 2016). Upon activation, cGMP production is increased and triggers the opening of cyclic nucleotide‐gated channels, such as TAX‐2 and TAX‐4, which depolarize the cells (Gray *et al*., 2004). A decrease in ambient O_2_ concentration inhibits AQR, PQR, and URX activity but activates the BAG sensory neurons. In BAG, the atypical sGC GCY‐31 and GCY‐33 are activated by low O_2_ concentration (Zimmer *et al*., 2009). Therefore, GCY‐31/GCY‐33 and GCY‐35/GCY‐36 display reciprocal responses to O_2_.

Previous studies showed that, in addition to sGC signaling, neuropeptide signaling plays a critical role in *C. elegans* O_2_ sensing (Gray *et al*., 2004). N2 worms that express the gain‐of‐function allele of neuropeptide receptor 1, *npr‐1(215V)*, do not avoid hyperoxia on food. However, animals bearing the weaker natural allele *npr‐1(215F)* or the loss‐of‐function *npr‐1(ad609)* allele show strong 21% O_2_ avoidance on food and aggregate on the bacterial lawn border, where there is a thicker growth of bacteria and, therefore, less O_2_ (~13%) (Gray *et al*., 2004). The aggregation of worms may further decrease the O_2_ concentration inside the clump and so create a preferable O_2_ concentration of around 8%. Therefore, N2 worms may experience higher O_2_ levels compared with *npr‐1(ad609)* animals under standard laboratory growth conditions. In this study, we explored the function of O_2_ sensing and neuropeptide signaling in lifespan regulation.

## Results

### NPR‐1 activity reduces *Caenorhabditis elegans* lifespan

**Figure 1:**
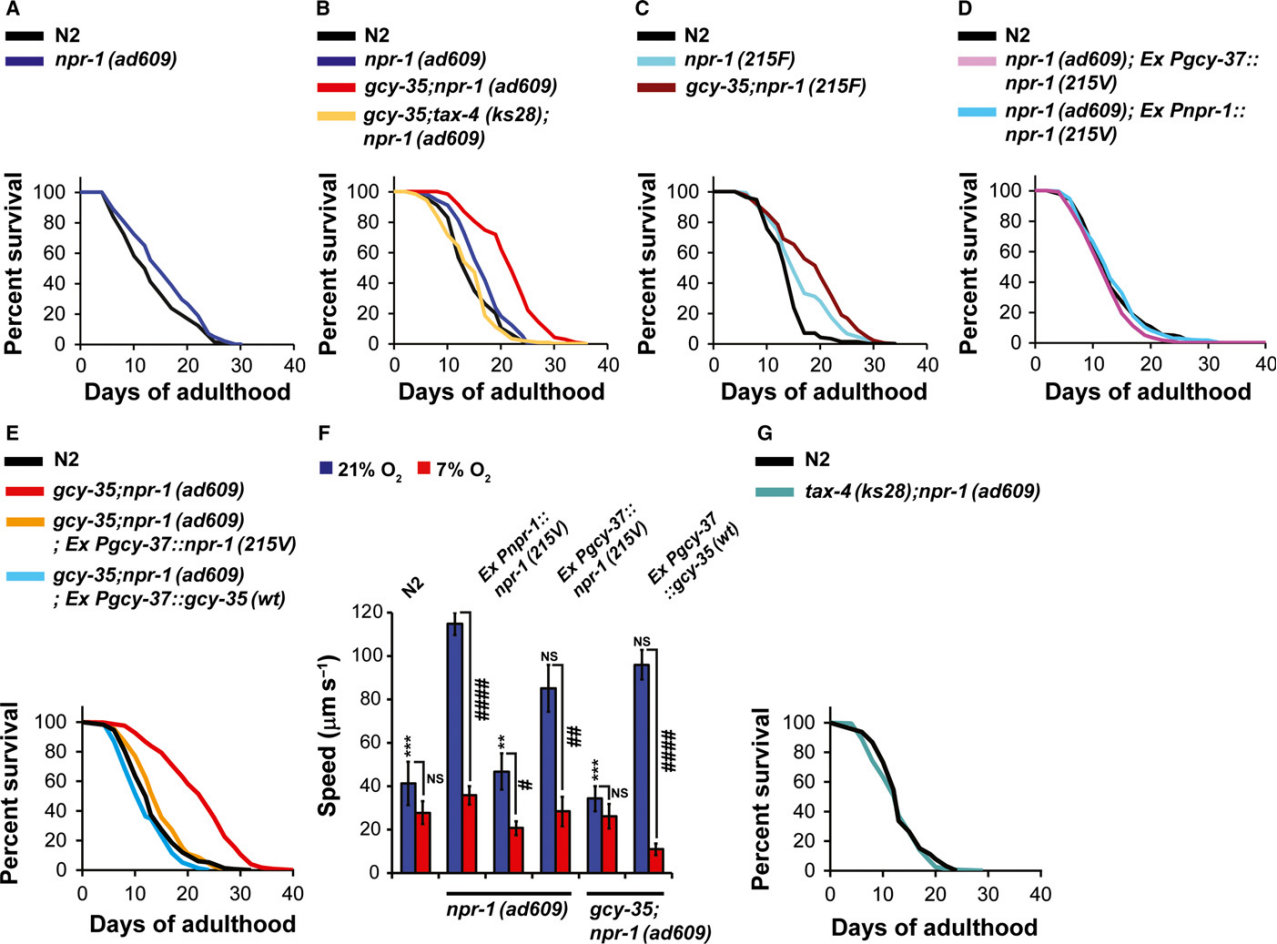
NPR‐1 and GCY‐35 regulate *Caenorhabditis elegans* lifespan. (A–E, G) Survival curves comparing the lifespans of worm strains. These experiments were performed at 21°C on live OP50. (F) Speed measurements. The speed of worms was measured at 21% and 7% O_2_ in the presence of OP50. Asterisks indicate significance for comparisons with *npr‐1* animals’ speed at 21% O_2_, Kruskal–Wallis test with Dunn's post‐test. Number sign indicates significance between speeds, within a strain. Unpaired t‐test with Welch's correction. *n *=* *6 or more assays performed over at least 3 days. ** *P *<* *0. 01, *** *P *<* *0.001, ##*P *<* *0. 01, ####*P *<* *0.0001, NS, nonsignificant, Error bars represent SEM.

The gain‐of‐function *npr‐1(215V)* allele appears to have arisen during domestication of the N2 strain to laboratory conditions (McGrath *et al*., 2009). As *npr‐1(215V)* activity suppresses the natural hyperoxia avoidance response on bacteria, we asked whether N2 worms suffer from misregulated O_2_‐homeostatic responses and therefore have a shorter lifespan than the aggregating strain *npr‐1(ad609)*. We measured the lifespans of N2 and *npr‐1(ad609)* worms on food. The lifespan of N2 worms was slightly but significantly shorter than that of *npr‐1(ad609)* animals (Fig. 1A; *P *= 0.0023), suggesting that NPR‐1(*215V*) activity is harmful to *C. elegans* longevity.

### Deletion of *gcy‐35* significantly increases *npr‐1(ad609)* worms’ lifespan

GCY‐35 activity is important for the O_2_ responses of *npr‐1(ad609)* animals (Gray *et al*., 2004). To explore whether GCY‐35 is also important for the extended lifespan of *npr‐1(ad609)* worms, we created an *npr‐1(ad609)* strain bearing the *gcy‐35(ok769)* deletion allele of *gcy‐35* and compared its lifespan to N2 and *npr‐1(ad609)* animals (Fig. 1B). The lifespan of *gcy‐35;npr‐1(ad609)* mutants was significantly longer than both N2 and *npr‐1(ad609)* worms, suggesting that the catalytic activity of GCY‐35 shortens the lifespan of *npr‐1(ad609)* worms.

We explored the lifespan of N2 worms bearing the *npr‐1(215F)* ancestral allele (this strain, QX1155 (McGrath *et al*., 2009), was kindly provided by the Bargmann laboratory) and of the *gcy‐35(ok769);npr‐1(215F)* double mutant. The lifespan of *npr‐1(215F)* worms was significantly longer than N2 controls (Fig. 1C, *P* < 0.0001), further supporting the conclusion that *npr‐1(215V)* activity shortens the lifespan of N2 worms. The deletion of *gcy‐35* significantly lengthened the lifespan of *npr‐1(215F)* worms (*P *= 0.0006), further supporting our conclusion that GCY‐35 activity shortens the lifespan of worms bearing either a nonfunctional or a weak allele of NPR‐1.

GCY‐35 and GCY‐36 are thought to form a functional heterodimer in AQR, PQR, and URX (Cheung *et al*., 2004; Zimmer *et al*., 2009). Therefore, *npr‐1(ad609) gcy‐36* mutants should have extended lifespan compared with N2 and *npr‐1(ad609)* worms. To test this, we crossed *npr‐1(ad609)* worms with worms bearing the *gcy‐36(ok2208)* loss‐of‐function allele and compared the lifespan of *npr‐1(ad609) gcy‐36* mutants to N2 and *npr‐1(ad609)* worms. Joint loss of function of *npr‐1* and *gcy‐36* significantly extended lifespan compared with both N2 and *npr‐1(ad609)* controls (Fig. S1A, Supporting information, *P *<* *0.0001 and *P *= 0.0034, respectively). As loss of function of either *gcy‐35* or *gcy‐36* extended the lifespan of *npr‐1(ad609)* worms and tracking the *gcy‐35* deletion was easier than tracking the *gcy‐36* deletion, we focused our studies on how combined loss of function of *gcy‐35* and *npr‐1* increases lifespan.

### NPR‐1 and GCY‐35 function in the AQR, PQR, and URX neurons to regulate lifespan

As both *npr‐1* and *gcy‐35* are expressed in the O_2_‐sensing neurons AQR, PQR, and URX, we hypothesized that the function of *npr‐1* and *gcy‐35* in these neurons decreases lifespan. To test this, we generated *npr‐1(ad609)* transgenes expressing genomic *npr‐1(215V)* only in AQR, PQR, and URX (using the *gcy‐37* promoter), or under its own promoter. In addition, we generated *gcy‐35;npr‐1(ad609)* transgenic animals expressing genomic *npr‐1(215V)* only in the AQR, PQR, and URX neurons, as well as *gcy‐35;npr‐1(ad609)* transgenes expressing *gcy‐35* cDNA in AQR, PQR, and URX. *npr‐1(ad609)* animals expressing *npr‐1(215V)* under its own promoter or the *gcy‐37* promoter had similar lifespans to N2 worms (Fig. 1D), indicating that *npr‐1(215V)* activity in the AQR, PQR, and URX neurons is essential and sufficient for shortening the extended lifespan of *npr‐1(ad609)* worms. Similarly, driving *npr‐1(215V)* expression only in the AQR, PQR, and URX neurons significantly shortened the lifespan of *gcy‐35;npr‐1(ad609)* mutants (Fig. 1E, *P* < 0.0001). Finally, restoring *gcy‐35* activity in AQR, PQR, and URX shortened the lifespan of *gcy‐35;npr‐1(ad609)* animals (Fig. 1E). Notably, a previous study by Powell‐Coffman and colleagues observed that *gcy‐37* is also expressed in AVM neurons and in two unidentified neurons in the head (Qin *et al*., 2006). However, we did not observe mCherry staining in AVM or any other neurons in the head beside AQR, and URX (see Fig. S1B, Supporting information). We performed two control experiments in which we used the *gcy‐34* promoter region to rescue the activity of *gcy‐35* and *npr‐1(215V)* in the AQR, PQR, and URX neurons of *gcy‐35;npr‐1(ad609)* mutants (*gcy‐34* is specifically expressed in AQR, PQR, and URX (Qin *et al*., 2006)). Similar to our previous results, expressing either *gcy‐35* or *npr‐1(215V)* under the promoter of *gcy‐34* significantly shortened the lifespan of *gcy‐35;npr‐1(ad609)* mutants (Fig. S1C, Supporting information), supporting the conclusion that *gcy‐35* and *npr‐1(215V)* act in AQR, PQR, and URX to shorten the lifespan of *gcy‐35;npr‐1(ad609)* mutants.

We verified the functionality of the *npr‐1* and *gcy‐35* expression constructs using speed measurements (Fig. 1F). Previous studies showed that *npr‐1(ad609)* worms move quickly on food at 21% O_2_ but sharply slow down at 7% O_2_ (Abergel *et al*., 2016), whereas N2 worms move slowly at both O_2_ concentrations (similar to *npr‐1(ad609)* worms at 7% O_2_). Moreover, the high foraging speed of *npr‐1(ad609)* worms on food requires *gcy‐35* activity (Cheung *et al*., 2005). Restoring *npr‐1(215V)* activity using its own promoter in *npr‐1(ad609)* worms reduced the speed of worms at 21% O_2_ to the low levels observed for N2 worms. *npr‐1(215V)* expression in AQR, PQR, and URX did not attenuate speed at 21% O_2_, but did suppress the extended lifespan of *gcy‐35;npr‐1(ad609)* worms (Fig. 1E), suggesting that *npr‐1* function in aging is not mediated by the hub‐neuron RMG (Macosko *et al*., 2009). Conversely, restoring *gcy‐35* activity in AQR, PQR, and URX in *gcy‐35;npr‐1(ad609)* worms significantly increased their speed at 21% O_2_ (Fig. 1F). Together, our data show that our rescuing constructs are functional and that the synergistic life extension of *gcy‐35;npr‐1(ad609)* worms depends on the combined loss of function of *npr‐1* and *gcy‐35* in AQR, PQR, and URX.

### TAX‐4 activity is important for the extended lifespan of *gcy‐35;npr‐1(ad609)* mutants

The activity of TAX‐2/TAX‐4 is important for cGMP signaling in some or all O_2_‐sensing neurons (Coates & de Bono, 2002; Zimmer *et al*., 2009). Indeed, previous studies showed that the *tax‐4(ks28)* strong loss‐of‐function allele suppresses GCY‐35 activity in AQR, PQR, and URX (Gray *et al*., 2004). Therefore, we hypothesized that joint loss of function of *tax‐4* and *npr‐1* would recapitulate the synergistic effect of *gcy‐35* and *npr‐1* loss of function on lifespan. However, our results showed that the lifespan of *tax‐4(ks28);npr‐1(ad609)* double mutants is similar to N2 worms (Fig. 1G). Moreover, we found that activity of TAX‐4 is required for the extended lifespan of *gcy‐35;npr‐1(ad609)* mutants, as the *gcy‐35;tax‐4(ks28);npr‐1(ad609)* triple mutants and N2 worms had similar lifespans (Fig. 1B, *P* = 0.8745).

A previous study showed that the *tax‐4* (*ky89)* and *tax‐4(p678*) loss‐of‐function alleles significantly lengthen the lifespan of N2 worms at 20°C (Apfeld & Kenyon, 1999). However, our experiments show that *tax‐4(ks28)* mutants and N2 worms have similar lifespans (*P *= 0.6112, Fig. S1D, Supporting information). Apart from using a different loss‐of‐function allele of *tax‐4*, a significant difference between our experiments and previous studies is that we did not use the thymidylate synthetase inhibitor FUDR in our lifespan assays. Therefore, we investigated whether the function of *tax‐4* in lifespan regulation is modulated by FUDR. FUDR significantly increased the lifespan of *tax‐4(ks28)* mutants compared with control *tax‐4(ks28)* mutants that grew without FUDR (*P *<* *0.0001, Fig. S1D, Supporting information). In addition, a significant lengthening of the lifespan of N2 worms was observed (Fig. S1E, Supporting information). However, the lifespans of *tax‐4(ks28)* mutants and N2 worms that grew on FUDR were not significantly different (*P *= 0.6091). Therefore, while our results show that FUDR significantly affects the function of *tax‐4* in lifespan regulation, they do not recapitulate previous results. As FUDR inhibits both DNA synthesis in worms and bacterial proliferation (Portal‐Celhay *et al*., 2012), we explored whether the effect of *tax‐4* on lifespan is modulated by bacterial viability. We repeated the lifespan experiments using UV‐killed bacteria as a food source. The lifespan of *tax‐4(ks28)* mutants that were grown on dead bacteria was similar to *tax‐4(ks28)* mutants that grew on live bacteria and FUDR (*P *= 0.0858), suggesting that FUDR extends the lifespan of *tax‐4(ks28)* mutants by suppressing bacterial toxicity. Finally, under this experimental condition the lifespan of N2 worms was significantly lengthened (*P *<* *0.0001) and was similar to the lifespan of *tax‐4(ks28)* (Fig. S1F, Supporting information), suggesting that *tax‐4* may be important in the defense mechanism against bacterial toxicity.

### GCY‐33 activity is essential for the extended lifespan of *gcy‐35;npr‐1(ad609)* animals

**Figure 2:**
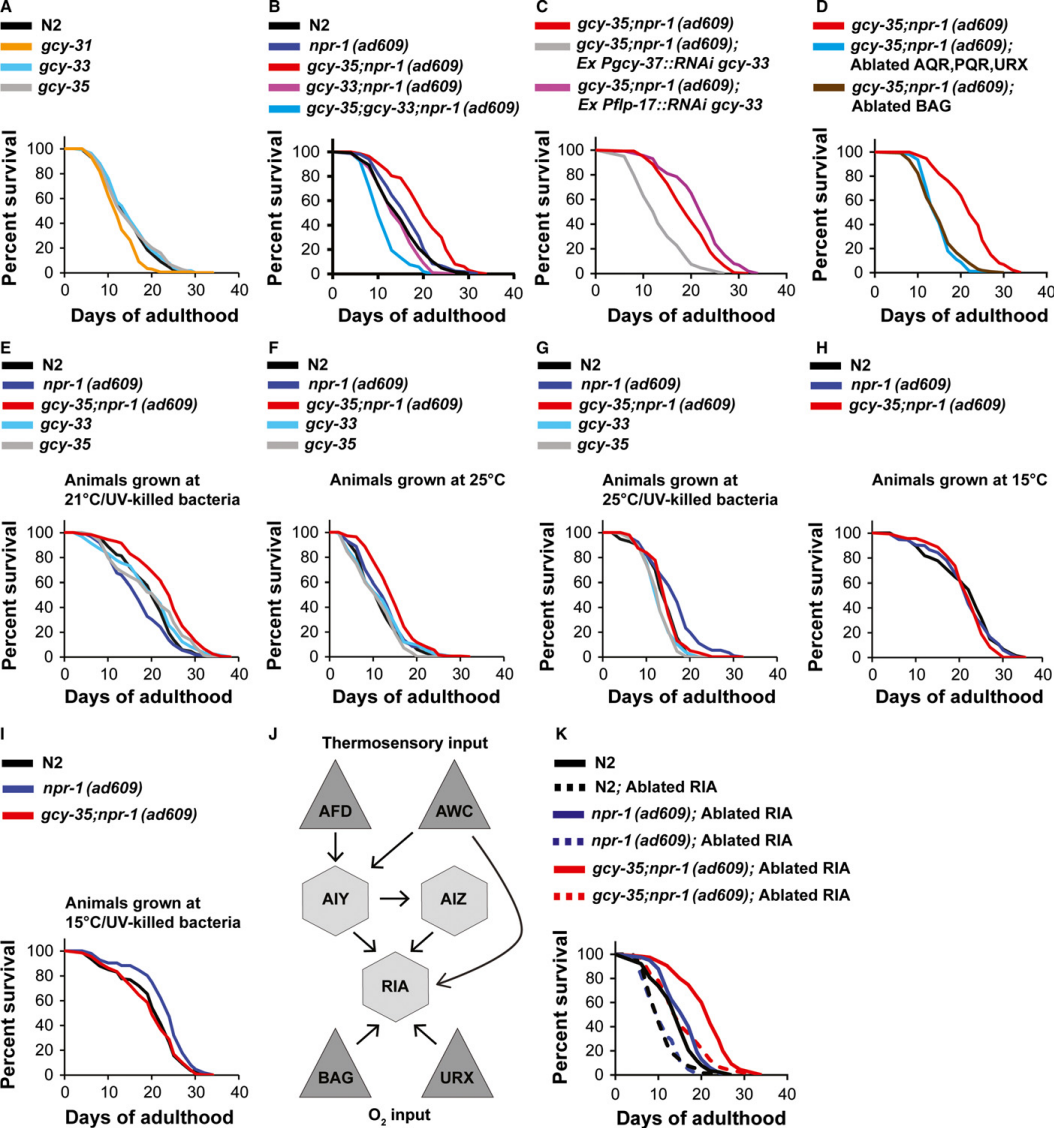
GCY‐31 and GCY‐33 are important for *gcy‐35;npr‐1(ad609)* animals’ extended lifespan. (A–D, K) Survival curves comparing the lifespans of worm strains at 21°C on live OP50. (C) Survival curves comparing the lifespan of *gcy‐35;npr‐1(ad609)* mutants to *gcy‐35;npr‐1(ad609)* transgenic worms expressing *gcy‐33* RNAi in AQR, PQR, and URX (under the *gcy‐37* promoter) or in BAG (under the *flp‐17* promoter). (E–I) Survival curves comparing the lifespans of worm strains at different growth conditions, as indicated by the labels above the graphs. (J) Circuit diagram of neurons with synaptic connections to RIA.

Our results show that the function of *tax‐4* is important for the extended lifespan of both *npr‐1(ad609)* and *gcy‐35;npr‐1(ad609)* worms. As the sGC *gcy‐33* and *tax‐4* function in URX and BAG (Gray *et al*., 2004; Zimmer *et al*., 2009), we next explored the function of *gcy‐33* in lifespan regulation. To explore whether *gcy‐33* regulates the lifespan of *npr‐1(ad609)* and *gcy‐35;npr‐1(ad609)* worms, we replaced the wild‐type allele of *gcy‐33* with the *gcy‐33(ok232)* deletion allele. The deletion of *gcy‐33* did not affect N2 worms’ lifespan (Fig. 2A). In contrast, *gcy‐33* deletion significantly shortened the lifespan of *npr‐1(ad609)* and *gcy‐35;npr‐1(ad609)* worms (Fig. 2B). Notably, the mean lifespan of *gcy‐35;gcy‐33;npr‐1(ad609)* mutants was significantly shorter than *npr‐1(ad609)* and *gcy‐33;npr‐1(ad609)* mutants (*P *<* *0.0001), suggesting a negative synergistic interaction between *gcy‐33* and *gcy‐35*.

Previous studies suggested that GCY‐31 and GCY‐33 form a functional O_2_‐sensor complex in BAG (Zimmer *et al*., 2009). To see whether deletion of *gcy‐31* and *gcy‐33* has a similar effect, we measured the lifespan of N2 worms bearing the *gcy‐31(ok296)* deletion allele. Unlike *gcy‐33* mutants, *gcy‐31* mutants had a significantly shorter lifespan than N2 animals (Fig. 2A). Next, we generated *gcy‐35;gcy‐31 npr‐1(ad609)* mutants and compared their lifespan to *gcy‐35;npr‐1(ad609)* worms. The lifespan of *gcy‐35;gcy‐31 npr‐1(ad609)* mutants was significantly shorter than *gcy‐35;npr‐1(ad609)* worms but significantly longer than *gcy‐35;gcy‐33;npr‐1(ad609)* mutants (*P *<* *0.0001, and *P *= 0.0137, respectively) (Fig. S2A, Supporting information), suggesting that the function of *gcy‐33* in lifespan regulation is not restricted to the GCY‐31/GCY‐33 complex.

### GCY‐33 activity in AQR, PQR, and URX is essential for lifespan extension of *gcy‐35;npr‐1(ad609)* animals

Unlike *gcy‐31*, the expression pattern of *gcy‐33* is not limited to BAG and includes the AQR, PQR, and URX neurons (Zimmer *et al*., 2009). To explore where *gcy‐33* function is important for lifespan regulation, we performed cell‐specific RNAi experiments. To knock down *gcy‐33* activity in BAG, we generated transgenes expressing *gcy‐33* RNAi under the *flp‐17* promoter. To knock down *gcy‐33* activity in AQR, PQR, and URX, we generated transgenic worms expressing *gcy‐33* RNAi under the *gcy‐37* promoter. Knocking down *gcy‐33* expression in the AQR, PQR, and URX neurons of *gcy‐35;npr‐1(ad609)* worms shortened the lifespan of these worms to below the level of N2 worms (*P *=* *0.0153; Fig. 2C), recapitulating the results obtained for the *gcy‐35;gcy‐33;npr‐1(ad609)* mutant (Fig. 2B). By contrast, *gcy‐35;npr‐1(ad609)* worms expressing *gcy‐33* RNAi only in BAG had a significantly longer lifespan than *gcy‐35;npr‐1(ad609)* controls (Fig. 2C, *P* = 0.0002). Knocking down *gcy‐33* expression in N2 worms, either in BAG or AQR, PQR, and URX did not have a significant effect on worm lifespan (Fig. S2B, Supporting information). Knocking down *gcy‐33* in AQR, PQR, and URX significantly shortened the lifespan of *npr‐1(ad609)* worms (Fig. 2SC, Supporting information); however, *gcy‐33* RNAi in BAG did not affect their lifespan. Together, our results suggest that the effect of *gcy‐33* activity on lifespan is cell specific and is dependent on the activity of *gcy‐35* and *npr‐1*.

### The O_2_‐sensing neurons AQR, PQR, URX, and BAG are important for the extended lifespan of *gcy‐35;npr‐1(ad609)* animals

Our results show that both *gcy‐31* and *gcy‐33* are essential for the extended lifespan of *gcy‐35;npr‐1(ad609)* mutants (Figs 2B and S2A, Supporting information). As *gcy‐31* is exclusively expressed in BAG, and the function of *gcy‐33* in AQR, PQR, and URX is essential for the lifespan extension of *gcy‐35;npr‐1(ad609)* worms, we hypothesized that all four O_2_‐sensing neurons are required for the lifespan extension of *gcy‐35;npr‐1(ad609)* mutants. To test this, we made strains expressing the death activator *egl‐1* only in AQR, PQR, and URX and strains expressing caspase 3 only in BAG (the parental strains for these crosses, CX7102 and CX11697, were kindly provided by the Bargmann laboratory). Ablation of AQR, PQR, and URX significantly shortened the lifespan of N2, *npr‐1(ad609)*, and *gcy‐35;npr‐1(ad609)* worms (Figs 2D and S2D,E, Supporting information). BAG ablation significantly shortened the lifespan of *gcy‐35;npr‐1(ad609)* and *npr‐1(ad609)* worms (Figs 2D and S2D, Supporting information, respectively) and, however, significantly increased the lifespan of N2 worms (Fig. S2E, Supporting information). Therefore, our experiments suggest that AQR, PQR, and URX are generally important for life extension and that BAG regulates lifespan in an *npr‐1*‐dependent manner.

### Temperature and food quality affect the function of *npr‐1* and *gcy‐35* in lifespan regulation

A previous study, conducted on the N2 background, showed that *gcy‐33* deletion extends lifespan, whereas deletion of *gcy‐35* shortens lifespan (Liu & Cai, 2013). However, in our experiments, the same *gcy‐33* and *gcy‐35* deletion alleles did not affect N2 worm lifespan significantly (Fig. 2A). As Liu & Cai used different experimental conditions from ours (our assays were performed on live OP50 bacteria at 21°C, whereas Liu & Cai's experiments used UV‐killed OP50 bacteria at 25°C), we asked whether temperature and bacterial viability could explain the difference between the results. We measured lifespan in three growth conditions: 21°C/UV‐killed OP50; 25°C/live OP50; and 25°C/UV‐killed OP50. All strains lived longest on UV‐killed OP50 at 21°C (Fig. 2E). These results support previous studies that showed that live OP50 shortens *C. elegans* lifespan (Garigan *et al*., 2002). Importantly, under this experimental condition, *gcy‐33* and *gcy‐35* mutants had similar lifespans to N2 controls. However, N2 worms lived significantly longer compared with *npr‐1(ad609)* worms (*P *=* *0.0098), suggesting that thriving on dead OP50 bacteria requires NPR‐1(*215V*) activity. Growing the worms on live OP50 at 25°C shortened the lifespan of all strains (Fig. 2F). The lifespans of *gcy‐33*,* gcy‐35*, and *npr‐1(ad609)* mutants grown in this environment were similar to N2 controls. Finally, *gcy‐33* and *gcy‐35* mutants had shorter lifespans than N2 controls on dead OP50 at 25°C (Fig. 2G). Notably, *gcy‐35;npr‐1(ad609)* worms lived longer (compared with the other strains) on live and dead OP50 at 21°C (Fig. 2B,E) and on live OP50 at 25°C (Fig. 2F). However, when grown at 25°C on dead OP50, their lifespan was similar to N2 animals (Fig. 2G). Intriguingly, in this growth condition, *npr‐1(ad609)* animals lived significantly longer compared with the other strains, indicating that *gcy‐35* activity is essential to thriving in this environment. Taken together, our results show that the synergistic effect of *npr‐1* and *gcy‐35* loss of function on lifespan is modulated by both temperature and bacterial viability. Moreover, the discrepancy between our data and that of and Liu & Cai could be attributed to experimental conditions in the case of *gcy‐35* mutants, as our findings at 25°C on dead OP50 are in agreement with theirs. However, the discrepancy between the *gcy‐33* mutant lifespan data could not be explained by differences in temperature or bacteria viability, as the *gcy‐33* deletion did not lengthen the lifespan of N2 worms (nor *npr‐1(ad609)* or *gcy‐35;npr‐1(ad609)* mutants) in any condition we tested.

### Joint loss of function of *gcy‐35* and *npr‐1* does not extend lifespan at 15°C

Our results show that joint loss of function of *gcy‐35* and *npr‐1* affects lifespan in a temperature‐ and food quality‐dependent way. To further explore this observation, we measured the lifespan of N2, *npr‐1(ad609)*, and *gcy‐35;npr‐1(ad609)* worms while feeding on either live or UV‐killed OP50 at 15°C. The three strains had similar lifespans at 15°C when grown on live OP50 (Fig. 2H). Notably, at 15°C the lifespans of N2 and *npr‐1(ad609)* worms were significantly increased (compared with 21°C, *P *<* *0.0001), but that of *gcy‐35;npr‐1(ad609)* animals was not affected, suggesting that low temperature may recapitulate the effect of *gcy‐35* and *npr‐1* joint loss of function on lifespan. The lifespan of *npr‐1(ad609)* worms on UV‐killed OP50 at 15°C was significantly longer than both N2 and *gcy‐35;npr‐1(ad609)* worms (Fig. 2I, *P* = 0.0002 and *P *=* *0.0003, respectively), further supporting our conclusion that *gcy‐35* and *npr‐1* function in lifespan regulation is modulated by temperature and bacterial viability.

### RIA is important for the extended lifespan of *gcy‐35;npr‐1(ad609)* mutants

Previous studies suggested that the RIA interneuron integrates information about temperature from the AFD and AWC neurons, and information about O_2_ from the URX and BAG neurons ((Kimata *et al*., 2012; Luo *et al*., 2014) for illustration, see Fig. 2J). Therefore, we hypothesized that RIA is important for the extended lifespan of *npr‐1(ad609)* and *gcy‐35;npr‐1(ad609)* mutants. To explore this, we genetically ablated RIA by expressing the death activator *egl‐1* with the promoter region of the RIA‐specific gene *glr‐3* (Brockie *et al*., 2001). The ablation of RIA shortened the lifespan of *gcy‐35;npr‐1(ad609)* mutants so it was similar to that of *npr‐1(ad609)* worms (*P *=* *0.6392, Fig. 2K), suggesting that RIA function is essential for the synergistic effect of *gcy‐35* and *npr‐1* loss of function on lifespan. Moreover, the lifespans of RIA genetically ablated N2 and *npr‐1(ad609)* transgenic worms were similar (*P *=* *0.6906) and significantly shorter than control worms (*P *<* *0.0001), suggesting that RIA is also important for the individual effect of *npr‐1* loss of function on lifespan. Notably, the lack of difference between the lifespans of N2 and *npr‐1(ad609)* worms with ablated RIA cannot be attributed to suppression of behavioral O_2_ responses, because the ablation of RIA did not reduce the accumulation of *npr‐1(ad609)* worms on the bacterial lawn border (Fig. S2F, Supporting information).

These results show that the RIA interneurons play an important role in lengthening the lifespan of *npr‐1(ad609)* and *gcy‐35;npr‐1(ad609)* worms and so suggest that the effect of joint loss of function of *npr‐1* and *gcy‐35* on lifespan regulation is mediated by neuronal communication.

### GCY‐35 and NPR‐1(*215V*) function in lifespan regulation is mediated by neurotransmitter/neuropeptide signaling

UNC‐31 regulates the release of neuropeptides from dense core vesicles (DCV) (Taylor & Dillin, 2013), and UNC‐13 and UNC‐64 are important for both neuropeptide and neurotransmitter release from DCV and small clear vesicles (Saifee *et al*., 1998; Sieburth *et al*., 2007). To explore whether neurotransmitter and/or neuropeptide signaling are important for the extended lifespan of *gcy‐35;npr‐1(ad609)* mutants, we made strains bearing the *unc‐13(e450)*,* unc‐31(e928),* and *unc‐64(e246)* loss‐of‐function mutations. The *unc‐13 gcy‐35;npr‐1(ad609)* and *gcy‐35;unc‐31;npr‐1(ad609)* mutants had significantly shorter lifespans than *gcy‐35;npr‐1(ad609)* worms (Fig. S3A, Supporting information). However, the *unc‐64* mutation did not significantly reduce the lifespan of *gcy‐35;npr‐1(ad609)* mutants. Therefore, although these results suggest that neuropeptide and/or neurotransmitter release is important for the lengthened lifespan of *gcy‐35;npr‐1(ad609)* mutants, they should be interpreted with caution. The *unc‐13* and *unc‐31* mutants had significantly longer lifespans than N2 worms (Fig. S3B, Supporting information). However, these mutations did not affect the lifespan of *npr‐1(ad609)* mutants (Fig. S3C, Supporting information), suggesting that the extended lifespan of *unc‐13* and *unc‐31* mutants requires the activity of NPR‐1(*215V*). The lifespan of *unc‐64* mutants was similar to N2 controls (Fig. S3B, Supporting information). However, *unc‐64;npr‐1(ad609)* mutants had a significantly shorter lifespan than both N2 and *npr‐1(ad609)* animals (*P *<* *0.0001, Fig. S3C, Supporting information), suggesting that the beneficial effect of *npr‐1(215V)* loss of function on worm lifespan is mediated through neuropeptide and/or neurotransmitter signaling.

### EGL‐3 is required for the extended lifespan of *gcy‐35;npr‐1(ad609)* mutants

**Figure 3:**
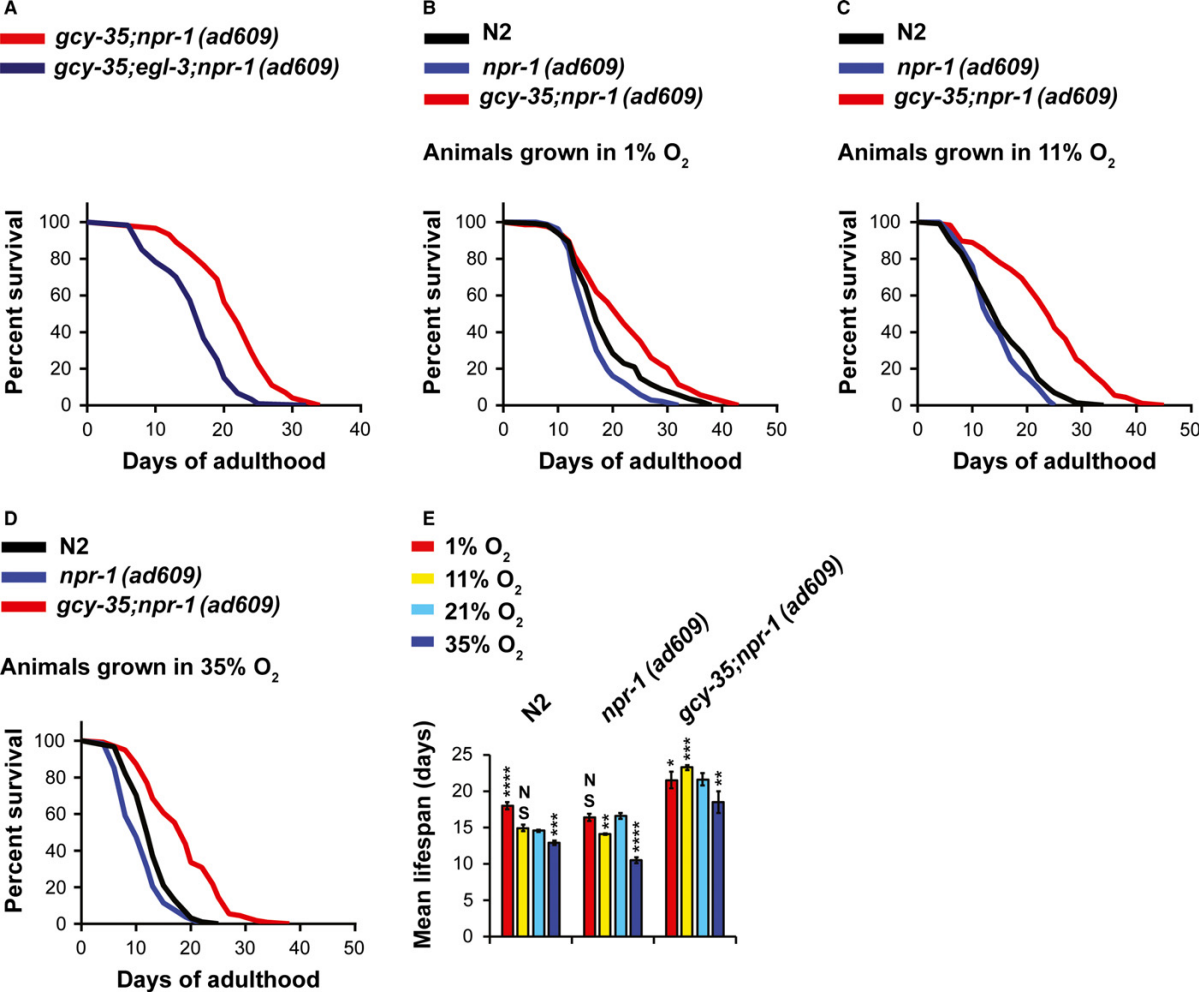
Neurotransmitter/neuropeptide signaling regulate the lifespan of *gcy‐35;npr‐1(ad609)* mutants. (A) Survival curves comparing the lifespans of worm strains at 21°C on live OP50 at 21% O_2_. (B–D) Survival curves comparing the lifespans of worm strains at different O_2_ levels, as indicated by the labels above the graphs. (E) Bar graph comparing mean lifespan at different O_2_ concentrations. The mean lifespan data for 1%, 11%, and 35% O_2_ were taken from C–E, respectively. Data for 21% O_2_ lifespan measurements were taken from Fig. 1B. Asterisks indicate significance for comparisons with N2's lifespan at each O_2_ level. *P <* *0.05, **P <* *0.01, ***P <* *0.001, ****P <* *0.0001, NS (not significant). Error bars represent SEM.

Our results suggest that joint loss of function of *npr‐1* and *gcy‐35* lengthens lifespan through neuropeptide signaling (Fig. S3A, Supporting information). The activity of the proprotein convertase EGL‐3 is important for transforming proneuropeptides into functional neuropeptides in *C. elegans* (Edwards *et al*., 2009). Therefore, we hypothesized that EGL‐3 is essential for the lengthened lifespan of *gcy‐35;npr‐1(ad609)* worms. To explore this, we created a *gcy‐35;npr‐1(ad609)* strain bearing the missense mutation allele *egl‐3(n150)*. The lifespan of *gcy‐35;egl‐3;npr‐1(ad609)* mutants was significantly shorter than *gcy‐35;npr‐1(ad609)* controls (Fig. 3A), suggesting that neuropeptide signaling is important for the lengthening of *gcy‐35;npr‐1(ad609)* mutant lifespan.

### NPR‐1(*215V*) regulates lifespan in an O_2_‐dependent manner

O_2_ regulates *C. elegans* lifespan. For example, exposure to 60% and 1% O_2_, respectively, shortens and lengthens the lifespan of N2 worms (Adachi *et al*., 1998). To explore the effect of O_2_ level on the lifespan of N2, *npr‐1(ad609)*, and *gcy‐35;npr‐1(ad609)* animals, we grew the worms at three O_2_ concentrations: 1%, 11%, and 35%. We chose these concentrations because 1% O_2_ induces hypoxic responses in *C. elegans* and was found to increase N2 lifespan (Adachi *et al*., 1998); 11% O_2_ inhibits high foraging speed and aggregation behavior of *npr‐1(ad609)* worms on food and is in the preferred O_2_ range for *C. elegans* (Gray *et al*., 2004); and 35% O_2_ may trigger oxidative stress but is still within the physiological range that nematodes experienced in the carboniferous period (~300 million years ago) (Halliwell & Gutteridge, 2006). In general, *gcy‐35;npr‐1(ad609)* mutants had a significantly longer lifespan at all tested O_2_ conditions than both N2 and *npr‐1(ad609)* animals (Fig. 3B–E), suggesting that the synergistic effect of *gcy‐35* and *npr‐1* loss of function on lifespan is O_2_ independent. By contrast, the effect of *npr‐1(215V)* on lifespan was O_2_ dependent (Fig. 3B–E). At 1% O_2_, N2 worms lived significantly longer than *npr‐1(ad609)* worms (Fig. 3B); at 11% O_2_, the lifespan of N2 and *npr‐1(ad609)* animals was similar (Fig. 3C); the lifespan of N2 worms at 35% was significantly longer than *npr‐1(ad609)* worms (Fig. 3D). In fact, only at 21% O_2_ was the lifespan of *npr‐1(ad609)* mutants longer than N2 worms. Therefore, we conclude that *npr‐1(215V)* effects lifespan in an O_2_‐dependent way.

### The extended lifespan of *npr‐1(ad609)* and *gcy‐35;npr‐1(ad609)* mutants cannot be explained by decreased metabolism

**Figure 4:**
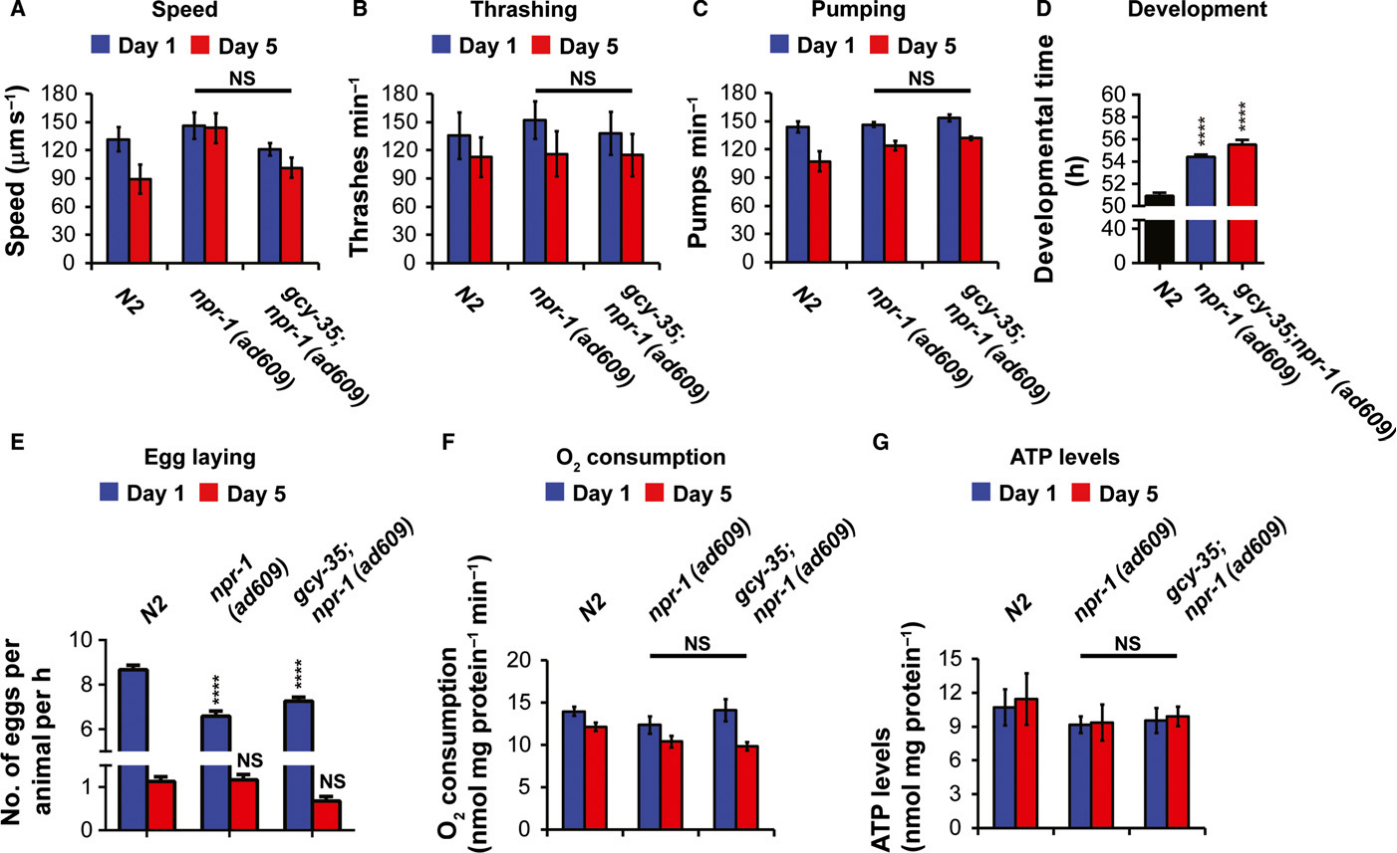
NPR‐1 and GCY‐35 effects on behavior, development, O_2_ consumption, and ATP levels. All experiments (apart from D) were performed on the first and fifth days of adulthood. (A, B) The speed and thrashing measurement were performed in the absence of bacteria. (C) Pharyngeal pumping behavior. (D) The development of worms was monitored from the L1 to the adult stage. Worms that had at least one egg in their bodies were counted as adults. Asterisks indicate significance for comparisons with N2 worms (one‐way ANOVA with Bonferroni post‐test). (E) Egg‐laying measurements. Asterisks indicate significance for comparisons with N2 worms at days 1 and 5 (two‐way ANOVA with Bonferroni post‐test). (F, G) O_2_ consumption and ATP measurements. One‐way ANOVA with Tukey's multiple comparisons post‐test. All assays were performed over the course of 3 days. The number of assays and the number of worms tested in each assay are indicated in Table S11 (Supporting information). Error bars represents SEM. ****P <* *0.0001, NS (not significant).

Some longed‐lived mutants, such as *clk‐1* and *daf‐2* (mutants with reduced mitochondrial activity and insulin signaling, respectively), have reduced metabolic rates compared with N2 worms (Van Voorhies & Ward, 1999). Therefore, we asked whether the extended lifespan of *gcy‐35;npr‐1(ad609)* mutants is associated with decreased metabolic activity. We measured the locomotory activity of worms on both solid medium and liquid medium (speed and thrashing assays respectively), feeding (pumping assays), development, egg laying rate, O_2_ consumption, and ATP levels (Fig. 4). These parameters (apart from development) were measured on the first and fifth days of adulthood, which precede the rapid decline of N2 worm viability. The locomotory activity and pharyngeal pumping of N2, *npr‐1(ad609)*, and *gcy‐35;npr‐1(ad609)* worms were similar on days 1 and 5 (Fig. 4A–C). By contrast, N2 worms developed slightly faster compared with the other strains (Fig. 4D) and had a higher egg laying rate on day 1, but not on day 5 (Fig. 4E). Notably, *npr‐1(ad609)* worms developed slightly faster than *gcy‐35;npr‐1(ad609)* worms, but laid fewer eggs on day 1. Therefore, although our results suggest that NPR‐1(*215V*) and GCY‐35 signaling is important for both development and egg‐laying regulation, the small differences between the strains probably cannot explain the lengthened lifespan of *gcy‐35;npr‐1(ad609)* mutants. To directly compare the metabolic activity of N2, *npr‐1(ad609)*, and *gcy‐35;npr‐1(ad609)* animals, we measured O_2_ consumption and ATP levels in the three strains (Fig. 4F,G). Consumption of O_2_ and ATP levels of the N2, *npr‐1(ad609)*, and *gcy‐35;npr‐1(ad609)* were similar on both days 1 and 5, indicating that the metabolic activity of these strains is similar.

### The canonical *daf‐2/daf‐16* pathway modulates *npr‐1*/*gcy‐35* function in lifespan regulation

**Figure 5:**
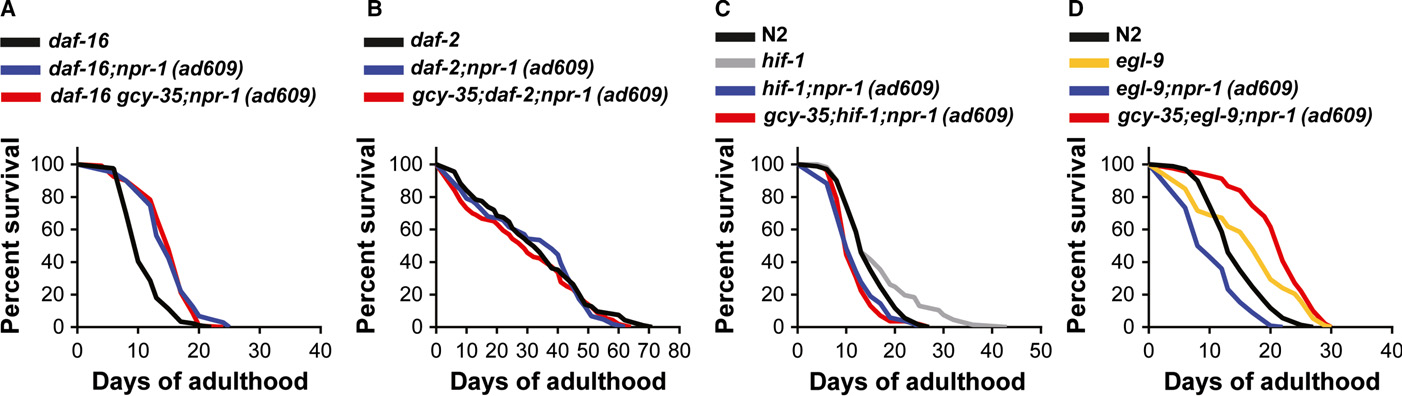
IIS and HIF‐1 signaling interact genetically with *npr‐1* and *gcy‐35*. (A–D) Survival curves comparing the lifespans of worm strains at 21°C on live OP50. One‐way ANOVA with Tukey's multiple comparisons post‐test. NS (not significant). Error bars represents SEM.

Our results suggest that reduction in metabolic rates cannot explain the extended lifespan of *gcy‐35;npr‐1(ad609)* mutants. Therefore, we set out to find the signaling pathway that lengthens the lifespan of *gcy‐35;npr‐1(ad609)* mutants. Insulin and insulin‐like growth factor signaling (IIS) plays a critical role in lifespan regulation (Murphy & Hu, 2013). In *C. elegans*, the activity of the insulin receptor DAF‐2 stops DAF‐16 (the *C. elegans* ortholog of the mammalian FOXO transcription factor) entering the nucleus. Therefore, when *daf‐2* is inhibited, DAF‐16 translocates to the nucleus and activates genes involved in stress resistance and longevity. In addition, DAF‐16 translocation to the nucleus is triggered by various environmental stimuli, including oxidative stress (Murphy & Hu, 2013). To explore whether IIS is important for *npr‐1/gcy‐35* signaling, we generated N2, *npr‐1(ad609)*, and *gcy‐35;npr‐1(ad609)* strains bearing the *daf‐16(mu86)* deletion allele and the *daf‐2(e1370)* loss‐of‐function allele and performed lifespan experiments. As expected, *daf‐16* significantly shortened the lifespan of the three strains (Fig. 5A). However, *daf‐16;npr‐1(ad609)* mutants lived significantly longer than *daf‐16* mutants (*P *<* *0.0001), indicating that the beneficial effect of NPR‐1*(215V*) inhibition is not mediated by *daf‐16* signaling. By contrast, the lifespan of *daf‐16;npr‐1(ad609)* mutants was similar to that of *daf‐16 gcy‐35;npr‐1(ad609)* mutants, indicating that the extended lifespan of *gcy‐35;npr‐1(ad609)* worms is dependent on DAF‐16 activity. The lifespan of *daf‐2(e1370)* mutants was not further extended by either *npr‐1* loss of function or the joint loss of function of *npr‐1/gcy‐35* (Fig. 5B), suggesting that lifespan lengthening by inhibition of *daf‐2* and *npr‐1/gcy‐35* takes place through overlapping mechanisms.

### HIF‐1 is essential for the extended lifespan of *gcy‐35;npr‐1(ad609)* mutants

Our results suggest that *npr‐1* and *gcy‐35* interact with the canonical IIS pathway. As the IIS pathway is important for life span extension in hypoxia (Leiser *et al*., 2013), we next explored whether the hypoxia‐inducible factor 1 (HIF‐1) is needed for the lifespan lengthening of *gcy‐35;npr‐1(ad609)* mutants. The transcription factor *hif‐1* plays a critical role in hypoxia signaling (Halliwell & Gutteridge, 2006). N2 worms bearing the *hif‐1(ia4)* deletion allele lived significantly longer than N2 controls (Fig. 5C). By contrast, the deletion of *hif‐1* shortened the lifespans of *npr‐1(ad609)* and *gcy‐35;npr‐1(ad609)* mutants to less than that of N2 (*P *<* *0.0001), suggesting that the beneficial effect of HIF‐1 on worm lifespan is mediated by NPR‐1(*215V*) activity. Moreover, our data suggest that the synergistic effect of *npr‐1/gcy‐35* loss of function on lifespan is mediated by HIF‐1.

HIF‐1 levels are regulated by the prolyl hydroxylase EGL‐9, which targets HIF‐1 for degradation. Therefore, inhibition of EGL‐9 results in accumulation of HIF‐1 even at 21% O_2_ (Zhang *et al*., 2009). To examine the effect of HIF‐1 accumulation on worm lifespan, we introduced the *egl‐9(sa307)* loss‐of‐function allele to N2, *npr‐1(ad609)*, and *gcy‐35;npr‐1(ad609)* worms and measured lifespan (Fig. 5D). *egl‐9* loss of function significantly increased the lifespan of N2 worms, but significantly shortened the lifespan of *npr‐1(ad609)* animals (Fig. 5D, *P* < 0.0001), indicating that the function of *egl‐9* in lifespan is regulated by *npr‐1*. Intriguingly, *egl‐9(sa307)* did not shorten the lifespan of *gcy‐35;npr‐1(ad609)* mutants, suggesting that the synergistic effect of *npr‐1/gcy‐35* on lifespan is not mediated by *egl‐9* function. In conclusion, our studies show that HIF‐1 function is required for the extended lifespan of *gcy‐35;npr‐1(ad609)* mutants (Fig. 5C). However, constitutive stabilization of HIF‐1 at 21% O_2_ is not required for the synergistic effect of *npr‐1/gcy‐35* loss of function on lifespan. *npr‐1* activity is essential for the beneficial effects of both *hif‐1* deletion and stabilization on lifespan.

### Are *gcy‐35;npr‐1(ad609)* mutants more resistant to stress?

**Figure 6:**
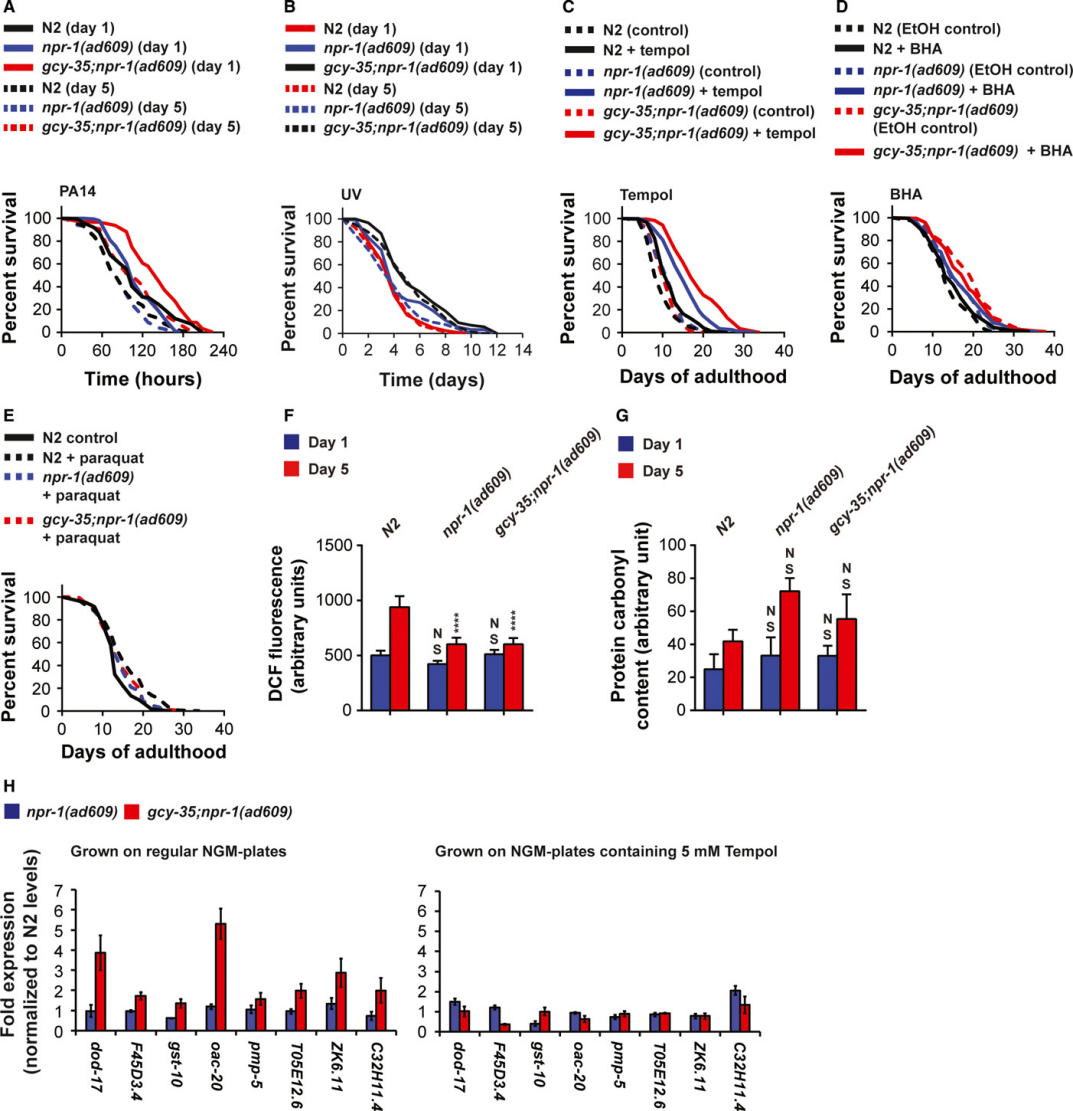
Survival assays of N2, *npr‐1(ad609)*, and *gcy‐35;npr‐1(ad609)* animals. (A) PA14 killing assays (B) Survival after UV exposure. The assays were performed on days 1 and 5 of adulthood. (C, D) Lifespan experiments in the presence of the antioxidants tempol (5 mM) and BHA (25 μm). (E) Lifespan experiments in the presence of 0.1 mm paraquat. (F) Bar graph comparing the levels of ROS in N2, *npr‐1(ad609)*, and *gcy‐35;npr‐1(ad609)* worms at days 1 and 5. Asterisks indicate significance for comparisons with N2 worms at days 1 and 5 (two‐way ANOVA with Bonferroni post‐test). (G) Protein oxidation measurements by Oxyblot. Quantification of Western blot analysis from at least four independent biological repeats, for each condition. Two‐way ANOVA with Bonferroni post‐test. (H) Gene expression measurements in N2, *npr‐1(ad609)*, and *gcy‐35;npr‐1(ad609)* worms that were either grown on regular NGM plates (left panel) or on NGM plates containing 5 mm Tempol (right panel). Gene expression was measured by qPCR. Each measurement represents, at least, three biological repeats.

The extended lifespan of *gcy‐35;npr‐1(ad609)* mutants is modulated by the activity of DAF‐16 and HIF‐1. As these transcription factors are important for protection against unfolded protein toxicity, heat, oxidative damage, and pathogenic bacteria (Back *et al*., 2012), we asked whether these animals live longer because they are more stress resistant. To address this question, we exposed N2, *npr‐1(ad609)*, and *gcy‐35;npr‐1(ad609)* worms on their first and fifth days of adulthood to various stresses and measured survival. Exposure to *Pseudomonas aeruginosa* PA14 bacteria induced rapid death in all three strains. However, the survival of *gcy‐35;npr‐1(ad609)* mutants was higher in both day 1 and day 5 of adulthood compared with N2 and *npr‐1(ad609)* worms (Fig. 6A), indicating that joint loss of function of *gcy‐35* and *npr‐1* provides protection against PA14 toxicity. Similarly, *gcy‐35;npr‐1(ad609)* mutants were more resistant to UV stress than N2 and *npr‐1(ad609)* worms (Fig. 6B). Interestingly, a previous study showed a striking similarity between the transcription of genes involved in DNA damage and innate immunity (Ermolaeva *et al*., 2013), suggesting that joint loss of function of *gcy‐35* and *npr‐1* induces an innate immunity response that extends the lifespan of *gcy‐35;npr‐1(ad609)* mutants. The increased resistance of *gcy‐35;npr‐1(ad609)* mutants to stress was specific to PA14 and UV. *gcy‐35;npr‐1(ad609)* mutants were significantly more sensitive to heat stress than N2 and *npr‐1(ad609)* worms on the first day of adulthood (Fig. S4A, Supporting information). However, on day 5 of adulthood the three strains showed similar survival rates, which were significantly higher than day 1, suggesting that older worms are generally more resistant to heat stress than young worms. Tunicamycin prevents the first step of N‐linked glycosylation of proteins in the ER so inducing accumulation of unfolded proteins and activating the unfolded protein response of the endoplasmic reticulum (UPR^ER^) (Taylor & Dillin, 2013). N2, *npr‐1(ad609)*, and *gcy‐35;npr‐1(ad609)* worms showed similar survival in response to tunicamycin (both on days 1 and 5 of adulthood, Fig. S4B, Supporting information). Finally, to explore whether *gcy‐35;npr‐1(ad609)* mutants are more resistant to mitochondrial oxidative stress, we exposed the three strains to 200 mM paraquat, a superoxide generator (Halliwell & Gutteridge, 2006), and measured their survival over 24 h. The survival of N2 and *npr‐1(ad609)* worms (on days 1 and 5) was similar to *gcy‐35;npr‐1(ad609)* mutants (Fig. S4C, Supporting information). Therefore, our results show that *gcy‐35;npr‐1(ad609)* mutants are not generally resistant to stress, but rather show specific resistance to both PA14 and UV toxicity.

### ROS is required for the extended lifespan of *gcy‐35;npr‐1(ad609)* mutants

As ROS signaling appears to be important for tolerance to UV and pathogenic bacteria (Hideg *et al*., 2013; Schieber & Chandel, 2014), we explored whether ROS is needed for the extended lifespan of *gcy‐35;npr‐1(ad609)* mutants, by performing lifespan experiments in the presence of antioxidants. We used the superoxide scavenger tempol (5 mm), and butylated hydroxyanisole (BHA). Tempol treatment significantly reduced the lifespan of N2, *npr‐1(ad609)*, and *gcy‐35;npr‐1(ad609)* worms (Fig. 6C), suggesting that either excessive scavenging of superoxide is generally damaging to *C. elegans* or long exposure to 5 mM tempol induces a toxic effect that is not associated with ROS. That being said, tempol treatment affected the three strains differently. The lifespans of *npr‐1(ad609)* and *gcy‐35;npr‐1(ad609)* mutants were similar, suggesting that ROS is required for the positive synergistic effect of *npr‐1* and *gcy‐35* loss of function on lifespan. However, *npr‐1(ad609)* mutants lived longer than N2 worms in the presence of tempol (Fig. 6C), suggesting that the effect of *npr‐1* on lifespan is not mediated by ROS. BHA significantly increased the lifespan of N2 worms (Fig. 6D, *P* = 0.0394) compared with N2 worms living on NGM plates with ethanol (the concentration of ethanol was similar in all plates). However, *npr‐1(ad609)* worms growing on BHA lived longer than N2 worms with BHA (*P *=* *0.0272). These results are in agreement with the tempol experiments that suggested that the effect of *npr‐1* on lifespan is not mediated by ROS. The lifespan of *gcy‐35;npr‐1(ad609)* animals growing with BHA was longer than the lifespan of *npr‐1(ad609)* worms with BHA. However, the difference was much smaller and barely reached significance (*P *=* *0.0438). Therefore, although the BHA results are not as conclusive as the tempol results, they do suggest that the synergistic effect of *gcy‐35/npr‐1* loss of function on lifespan in regulated by ROS.

### Paraquat extends the lifespan of N2 worms but reduces the lifespan of both *npr‐1(ad609)* and *gcy‐35;npr‐1(ad609)* mutants

Previous studies showed that low levels of the superoxide generator paraquat lengthen the lifespan of N2 worms (Yang & Hekimi, 2010). Intriguingly, either a small increase or decrease in paraquat concentration can diminish the beneficial effect, suggesting that ROS act within a narrow range of concentrations to lengthen lifespan. Our studies suggest that the extended lifespan of *gcy‐35;npr‐1(ad609)* mutants is mediated by ROS. Therefore, we hypothesized that low levels of paraquat will not further extend the lifespan of *gcy‐35;npr‐1(ad609)* mutants and may even induce a toxic effect. We measured the lifespan of N2, *npr‐1(ad609)*, and *gcy‐35;npr‐1(ad609)* worms grown on NGM plates supplemented with 0.1 mM paraquat, a concentration shown to maximize the lifespan extension of N2 worms (Yang & Hekimi, 2010). As expected, paraquat treatment lengthened the lifespan of N2 worms significantly (Fig. 6E, *P* < 0.0001), supporting the results from previous studies. By contrast, it shortened the lifespans of *npr‐1(ad609)* and *gcy‐35;npr‐1(ad609)* mutants to N2 control level, suggesting that the level of ROS in these worms exceeded the beneficial level and elicited a toxic effect. Together, these results further support the hypothesis that ROS act within a narrow range of concentrations to lengthen worm lifespan and that excessive ROS is damaging.

### Accumulation of ROS and protein oxidation are not correlated with the extended lifespan of *gcy‐35;npr‐1(ad609)* mutants

To explore the metabolism of ROS and its consequences, we measured the levels of ROS and protein carbonyl oxidation in N2, *npr‐1(ad609)*, and *gcy‐35;npr‐1(ad609)* worms. To measure the overall level of ROS, we used the 2′,7′dichlorofluorescin diacetate (DCFDA) fluorescent dye. We measured ROS levels in N2, and *npr‐1(ad609)*, and *gcy‐35;npr‐1(ad609)* worms on the first and fifth days adulthood. The levels of ROS in all three trains were similar on day 1 (Fig. 6F), and also similar in *npr‐1(ad609)* and *gcy‐35;npr‐1(ad609)* mutants on day 5. However, the levels of ROS on day 5 were significantly higher in N2 worms compared with both *npr‐1(ad609)* and *gcy‐35;npr‐1(ad609)* mutants (*P *<* *0.001). Nevertheless, the conclusion from these studies is that ROS levels do not correlate with extended lifespan of *gcy‐35;npr‐1(ad609)* mutants. As degradation of ROS could be different in N2, *npr‐1(ad609)*, and *gcy‐35;npr‐1(ad609)* worms, we measured the oxidation of proteins on the first and fifth days of adulthood. Protein oxidation, as monitored by carbonyl accumulation, was similar in all strains on days 1 and 5 (Figs 6G and S4D, Supporting information), suggesting that ROS do not accumulate to levels that cause this commonly used gauge of protein oxidation.

In conclusion, our experiments failed to detect any correlation between the extended lifespan of *gcy‐35;npr‐1(ad609)* mutants and ROS levels or protein oxidation. However, our antioxidant, paraquat, DAF‐16, and HIF‐1 experiments strongly suggest that ROS is important for the extended lifespan of *gcy‐35;npr‐1(ad609)* mutants. A potential explanation for this discrepancy is that ROS are needed at small quantities in specific cells (*e.g*., the O_2_‐sensing neurons). However, future studies are needed to explore this hypothesis.

### The transcription of innate immunity genes in *gcy‐35;npr‐1(ad609)* mutants is ROS dependent

ROS controls the function of DAF‐16 and HIF‐1 and the activity of these transcription factors is important for both *gcy‐35;npr‐1(ad609)* life extension (Fig. 5) and innate immunity (Singh & Aballay, 2009; Hwang *et al*., 2014). Moreover, a previous study showed that innate immunity genes are differentially expressed in *gcy‐35;npr‐1(ad609)* mutants and *npr‐1(ad609)* mutants (Styer *et al*., 2008). Therefore, we asked whether ROS regulates the expression of innate immunity genes in *gcy‐35;npr‐1(ad609)* mutants. We extracted RNA from N2, *npr‐1(ad609)*, and *gcy‐35;npr‐1(ad609)* worms that were grown throughout their life cycle on either regular NGM plates (as controls) or NGM plates containing 5 mm tempol (experimental group) and measured the expression of eight genes known to be involved in the innate immunity (Engelmann *et al*., 2011; Gaglia *et al*., 2012; Twumasi‐Boateng & Shapira, 2012), using reverse‐transcription quantitative PCR (RT–qPCR). As shown in Fig. 6H (left panel), all eight genes (in the control group) were upregulated in *gcy‐35;npr‐1(ad609)* mutants compared with N2 and *npr‐1(ad609)* worms. However, in the presence of tempol the expression level of these genes was substantially downregulated in *gcy‐35;npr‐1(ad609)* mutants (Fig. 6H, right panel), indicating that the induction of these genes in *gcy‐35;npr‐1(ad609)* mutants is regulated by ROS.

## Discussion

### ROS signaling is essential for the extended lifespan of *gcy‐35;npr‐1(ad609)* animals

Harman's theory of aging (Harman, 1956) suggested that the creation of ROS *via* aerobic respiration underlies the development and progression of aging. However, studies from the last decade have challenged this theory. For example, *C. elegans* lacking all superoxide dismutases (*sod‐1*‐*sod‐5*) live longer than N2 animals, and chemical agents that enhance mitochondrial ROS increase lifespan (Van Raamsdonk & Hekimi, 2012). Our results suggest that tight regulation of ROS level is essential for the extended lifespan of *gcy‐35;npr‐1(ad609)* mutants (Fig. 6). Both ROS scavenging by antioxidants and increased ROS by sublethal level of paraquat shorten the lifespan of *gcy‐35;npr‐1|(ad609)* worms. This hypothesis is further supported by the requirement of DAF‐16 and HIF‐1 for the extended lifespan of *gcy‐35;npr‐1(ad609)* animals (Fig. 5), as these transcription factors are regulated by ROS (Hwang & Lee, 2011; Zou *et al*., 2013). Notably, the interpretation of our results should be made with caution, because we failed to detect any significant difference in either ROS accumulation or oxidative damage in *gcy‐35;npr‐1(ad609)* worms compared with both N2 and *npr‐1(ad609)* animals. That being said, we suspect that ROS act in specific neurons to regulate lifespan. Therefore, more specific and sensitive methods are needed to detect these localized changes.

Despite the above caveats, we suggest the following working model. The function of NPR‐1(*215V*) and GCY‐35/GCY‐36 in the O_2_‐sensing neurons AQR, PQR, and URX inhibits lifespan extension. By contrast, GCY‐33 signaling through TAX‐2/TAX‐4 triggers the secretion of neuropeptide/neurotransmitter that increases the level of ROS in either the O_2_‐sensing neurons themselves, downstream neurons, or even non‐neuronal tissues. The increase in ROS levels activates the HIF‐1 and DAF‐16 transcription factors which induce innate immunity. This induction of innate immunity increases lifespan. Although much of the details in this model are still not known, for example, the identity of its interacting partner of GCY‐33 in AQR, PQR, and URX, and where DAF‐16 and HIF‐1 function is needed for the life extension, this model provides a framework for future studies to identify the ‘anti‐aging’ neurotransmitter/neuropeptide and to elucidate how innate immunity genes prolong lifespan. In this respect, it is interesting to note that a previous study showed that the expression of the innate immunity genes *dod‐17*,* C32H11.4*, and *ZK6.11* is substantially downregulated in *daf‐10(m79)* worms (Gaglia *et al*., 2012). Moreover, the high sensitivity of *daf‐10(m79)* animals to PA14 is rescued by the *gcy‐35(ok769)* mutation. DAF‐10 is important for the activity of ciliated neurons, such as AQR, PQR, and BAG. Therefore, in the future it will be intriguing to explore whether *daf‐10* function in AQR, PQR, and BAG is important for the extended lifespan of *gcy‐35;npr‐1(ad609)* mutants and whether this function is ROS dependent.

## Experimental procedures

We used standard molecular biology and genetic protocols. A detailed description of strains and oligonucleotides used is found in Table S10 (Supporting information) and Supplemental Experimental Procedures.

### Lifespan analysis

All lifespan assays were conducted at 21°C and started with synchronized young adults, unless otherwise mentioned. Statistical analysis on lifespan survival curves was performed using the log‐rank (Mantel–Cox) test with Prism 6 software, and the results are displayed in Tables S1–S9 (Supporting information). Further details are found in Supplemental Experimental Procedures.

## Funding info

Research leading to these results received funding from the Marie Curie Career Integration Grants (CIG), agreement no. PCIG11‐GA‐2012‐322003. (L.L and R.A).

## Conflict of interest

The authors declare that they have no conflicts of interest.

## Author contributions

R.A., and L.L., and E.G. designed research; R.A., L.L., M.S., and A.K.C. performed research; R.A., L.L., and E.G. analyzed data; and R.A., L.L., and E.G. wrote the paper.

## Supporting information


**Fig. S1** The effect of GCY‐36 and TAX‐4 on N2 and *npr‐1(ad609)* worms’ lifespan (related to Figure 1).

**Fig. S2** GCY‐33 is important for *npr‐1(ad609)* animals’ extended lifespan (related to Figure 2).

**Fig. S3** The function of NPR‐1 in lifespan regulation is modulated by neuropeptide/neurotransmitter signaling (related to Figure 3).

**Fig. S4** Joint loss‐of‐function of *npr‐1* and *gcy‐35* does not increase tolerance to heat, ER UPR, or mitochondrial UPR stress (related to Figure 6).

**Table S1** Numerical values for data plotted in Fig. 1.

**Table S2** Numerical values for data plotted in Fig. S1.

**Table S3** Numerical values for data plotted in Fig. 2.

**Table S4** Numerical values for data plotted in Fig. S2.

**Table S5** Numerical values for data plotted in Fig. S3.

**Table S6** Numerical values for data plotted in Fig. 3.

**Table S7** Numerical values for data plotted in Fig. 5.

**Table S8** Numerical values for data plotted in Fig. 6.

**Table S9** Numerical values for data plotted in Fig. S4.

**Table 10** List of primers used for *npr‐1*,* gcy‐35* and *egl‐1* sequencing, primers for cell specific RNAi by PCR fusion, and quantitative RT‐PCR experiments.

**Table S11** Number of animals and experimental data.

**Appendix S1** Supplemental Experimental Procedures.
