# Supplementary figures and images for: Synergism between soluble guanylate cyclase signaling and neuropeptides extends lifespan in the nematode Caenorhabditis elegans
Source: Aging Cell. 2017 Jan 4;16(2):401–13. doi: 10.1111/acel.12569 (PMC5334569; doi:10.1111/acel.12569)

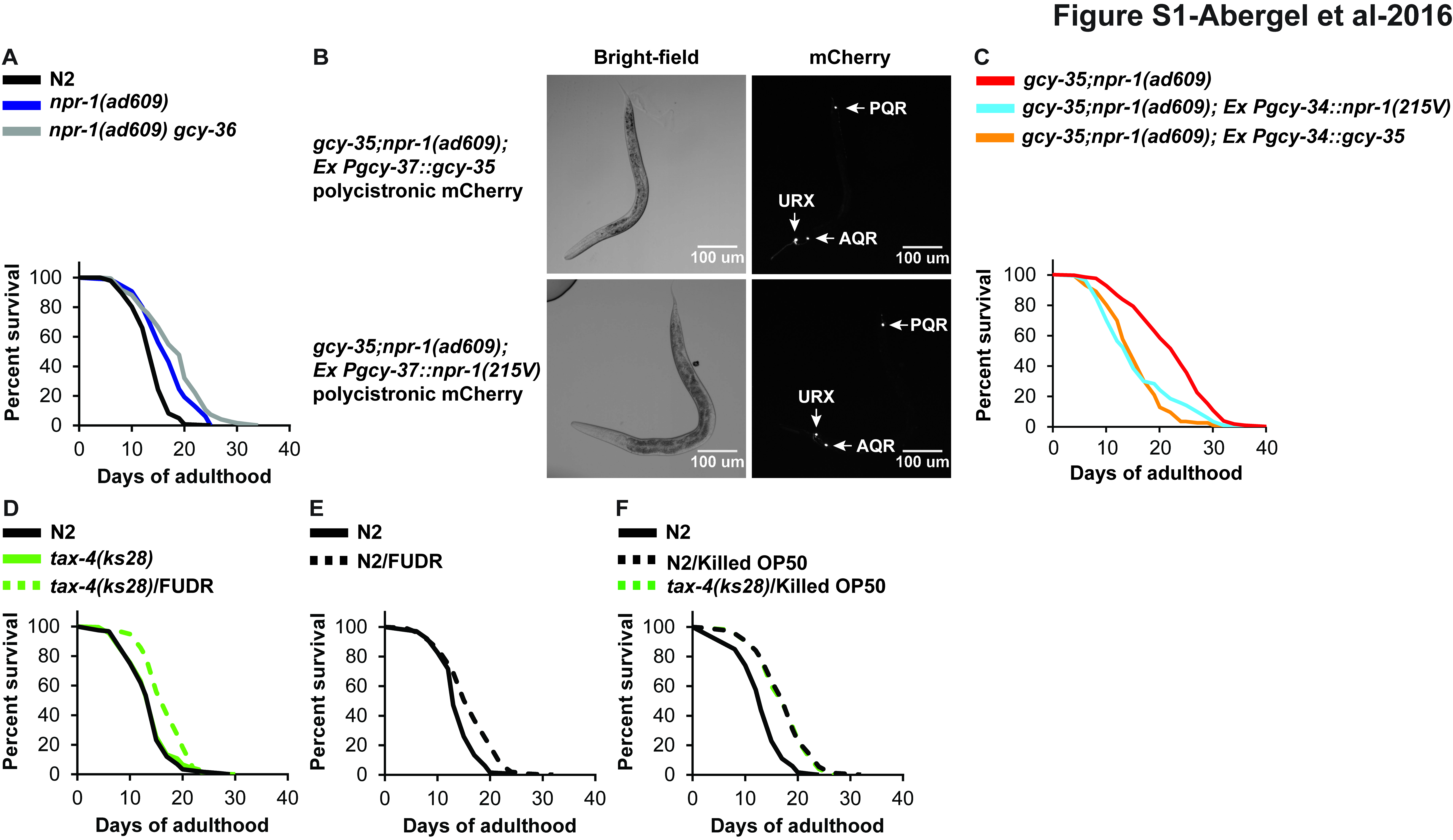

Supplement: Supplementary file 1 — Fig. S1 The effect of GCY‐36 and TAX‐4 on N2 and npr‐1(ad609) worms’ lifespan (related to Figure 1). [file ACEL-16-401-s001.tif]

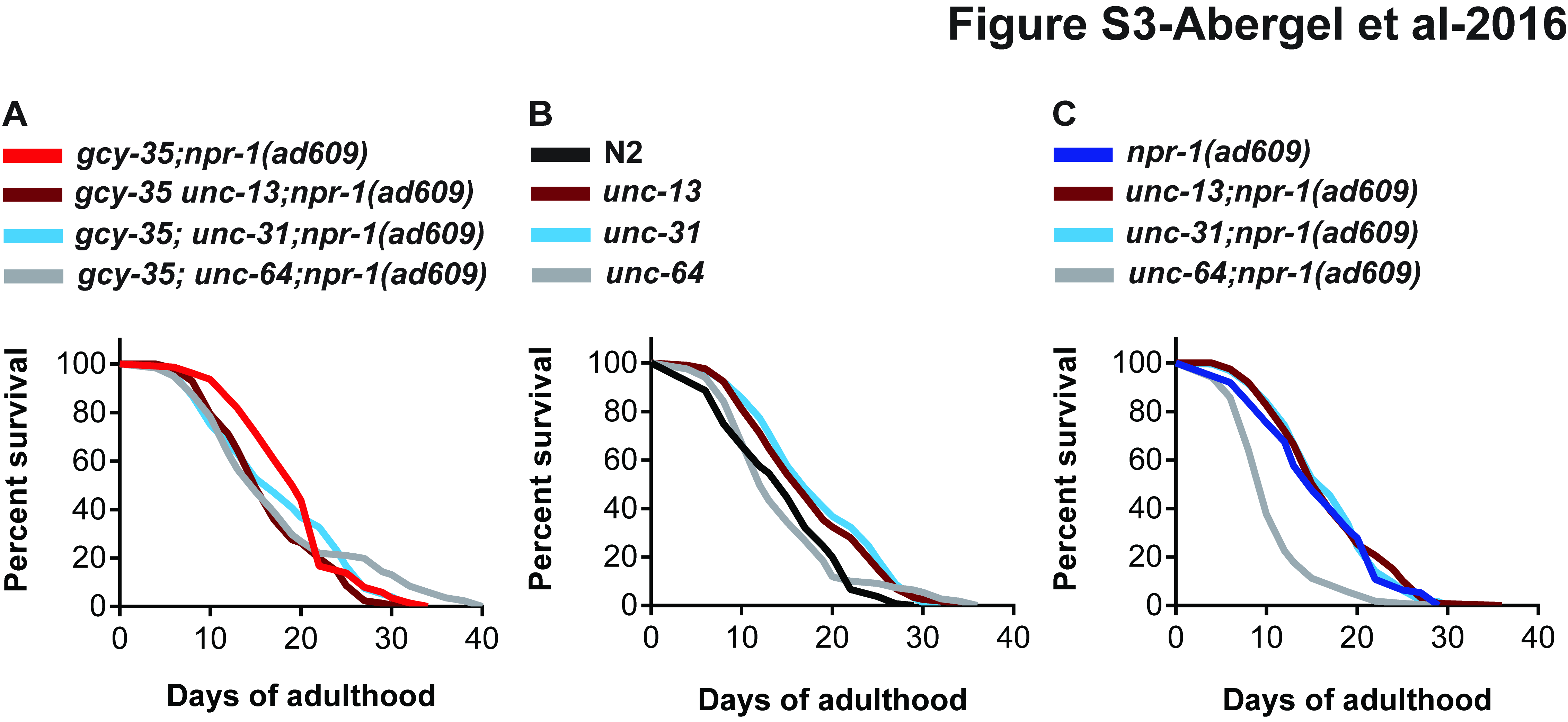

Supplement: Supplementary file 3 — Fig. S3 The function of NPR‐1 in lifespan regulation is modulated by neuropeptide/neurotransmitter signaling (related to Figure 3). [file ACEL-16-401-s003.tif]
