# Supplementary material for: Synergism between soluble guanylate cyclase signaling and neuropeptides extends lifespan in the nematode Caenorhabditis elegans
Source: Aging Cell. 2017 Jan 4;16(2):401–13. doi: 10.1111/acel.12569 (PMC5334569; doi:10.1111/acel.12569)
Supplement: Supplementary file 14 — Table 10 List of primers used for npr‐1, gcy‐35 and egl‐1 sequencing, primers for cell specific RNAi by PCR fusion, and quantitative RT‐PCR experiments. [file ACEL-16-401-s014.docx]

**Supplementary Table 10.** List of primers used for *npr-1*, *gcy-35* and *egl-1* sequencing, primers for cell specific RNAi by PCR fusion, and quantitative RT-PCR experiments.

| **Primers for *npr-1* sequencing** | | |
| --- | --- | --- |
| *npr-1* A R | 5’ TTAGCCGGGTGTCAGTAAGC 3’ | |
| *npr-1* B F | 5’ GCGATAGTGCCATTTCTGAC 3’ | |
| *npr-1* C F | 5’ GAACCTTCACTTCTCCTGTG 3’ | |
| *npr-1* D F | 5’ CTAGTACCCTCGTTTCAGGA 3’ | |
| *npr-1* E F | 5’ CCGACTTTATGGCAGAGATG 3’ | |
| *npr-1* F F | 5’ CGGGAAAGTGGCTAAAGTTG 3’ | |
| *npr-1* G F | 5’ GCACTTGAATGGAAGAGCCA 3’ | |
| **Primers for *gcy-35* sequencing** | | |
| 35Ex910 R | 5’ GTGATCGGATGTGATGACTGCCGGG 3’ | |
| 35Ex910 F | 5’ GGCGAACAAACAATCGACGGATATATGG 3’ | |
| 35F30500 | 5’ CTCGAAGCGCAGTGGTTTATATCCTATTG 3’ | |
| gcy35R600 | 5’ GTGAGTCAGCTTTTGAAGAAATCGATTTTGGC 3’ | |
| gcy35F750 | 5’ CCAAATGCGATGCGCCAAGAGACAC 3’ | |
| **Primers for *egl-1* sequencing** | | |
| *egl-1* F seq | 5' CTCTTCGGATCTTCTACCAATG 3' | |
| *egl-1* R seq | 5' GATCAGCTTTCATAATTTTGACAA C 3' | |
| **Primers for cell specific RNAi by PCR fusion** | | |
| A P*flp-17* | 5' GCTCTGACGTCACAGGCTTTAAAC 3' | |
| Ap P*flp-17* | 5' CGCATATTTTCCTTGAAGCTTTTCCTC 3' | |
| B AS P*flp-17-gcy-33* | 5' GCACATTCTCATTCAATCAGGAGCAAGCTGGAAAAATAAAGTTTTGCGG AAAATATTTCC 3' | |
| B AS P*gcy-37-gcy-33* | 5' GCACATTCTCATTCAATCAGGAGCAAGATTTCTGTGTAGTAGAAAAAGT AGAAAAGCG 3' | |
| B P*flp-17-gcy-33* | 5' CACCTTCAATGACCAATCCGTACATCTGGAAAAATAAAGTTTTGCGGAA AATATTTCC 3' | |
| B P*gcy-37-gcy-33* | 5' CACCTTCAATGACCAATCCGTACATATATTTCTGTGTAGTAGAAAAAGT AGAAAAGCG 3' | |
| C *gcy-33* cDNA | 5' ATGTACGGATTGGTCATTGAAGGTGTTCG 3' | |
| D *gcy-33* cDNA | 5' CTTGCTCCTGATTGAATGAGAATGTGC 3' | |
| Dp *gcy-33* cDNA | 5' ATGTACGGATTGGTCATTGAAGGTGTTCG 3' | |
| Dp *gcy-33* cDNA | 5' GTCGTTGTAGGTTTTGCAGACACAGTTG 3' | |
| P*gcy-37* A | 5' CGTAGAGGGACCGACATGCTCG 3' | |
| P*gcy-37* Ap | 5' GAGGGACCGACATGCTCGGCTTG 3' | |
| **Primers for qPCR** | | |
| **Name of the gene** | **Forward** | **Reverse** |
| *C32H11.4* | 5’ CTCTCCAGCCGATCCAAAT 3’ | 5’ GTTTCATAGAGCGTGTACAAG 3’ |
| *gst-10* | 5’ CAAGAGATTGTGCAGACTGG 3’ | 5’ CATGTCGAGGAAGGTTGCC 3’ |
| *dod-17* | 5' GGAAGAAGCTTGAGTTCAGC 3' | 5' GAGACTAGCTTCAAGGTTCAC 3' |
| *F45D3.4* | 5' CAATCCAGCTGCCACTTATC 3' | 5' CGTCGTTTGGAATTGGTCC 3' |
| *oac-20* | 5' GAATGGAAGGAAACTCTACTC 3' | 5' GAATATCCAAAGAGTGCAACC 3' |
| *pmp-5* | 5' CTCCGATCTTTCCTTCGATG 3' | 5' GAAGGATGTAGGACATGCAG 3' |
| *T05E12.6* | 5' GTTCAGGTAACAGATCAGCTC 3' | 5' CGATTTCCAGTCGAGAGTTG 3' |
| *ZK6.11* | 5' CTGTGGTCACTGAAAGTGG 3' | 5' GTTGTCGTAAGATAGGCAGG 3' |
| *ned-8* | 5’ CGAACCAAATGATCGAGTCG 3’ | 5’ CTTGTAATCTGCTGCTGTCTTG 3’ |
| *Y54G2A.18* | 5’ CACCTTCACCCTCCTTCTTC 3’ | 5’ GAGGATGAAGAGCACGAAGC 3’ |
