# Supplementary material for: Synergism between soluble guanylate cyclase signaling and neuropeptides extends lifespan in the nematode Caenorhabditis elegans
Source: Aging Cell. 2017 Jan 4;16(2):401–13. doi: 10.1111/acel.12569 (PMC5334569; doi:10.1111/acel.12569)
Supplement: Supplementary file 15 — Table S11 Number of animals and experimental data. [file ACEL-16-401-s015.docx]

| **Figure** | **Strain** | **Number of worms** | | **Number of experiments** | |
| --- | --- | --- | --- | --- | --- |
|  |  | Day 1 | Day 5 | Day 1 | Day 5 |
| **4A** | N2 | >80 | >64 | 10 | 8 |
|  | *npr-1(ad609)* | >104 | >88 | 13 | 11 |
|  | *gcy-35;npr-1(ad609)* | >72 | >64 | 9 | 8 |
| **4B** | N2 | 27 | 27 | 3 | 3 |
|  | *npr-1(ad609)* | 27 | 27 | 3 | 3 |
|  | *gcy-35;npr-1(ad609)* | 27 | 27 | 3 | 3 |
| **4C** | N2 | 32 | 30 | 3 | 3 |
|  | *npr-1(ad609)* | 30 | 30 | 3 | 3 |
|  | *gcy-35;npr-1(ad609)* | 30 | 30 | 3 | 3 |
| **4E** | N2 | 32 | 20 | 3 | 3 |
|  | *npr-1(ad609)* | 22 | 20 | 3 | 3 |
|  | *gcy-35;npr-1(ad609)* | 32 | 24 | 3 | 3 |
| **4F** | N2 | ~1200 | ~900 | 4 | 3 |
|  | *npr-1(ad609)* | ~1500 | ~900 | 5 | 3 |
|  | *gcy-35;npr-1(ad609)* | ~900 | ~900 | 3 | 3 |
| **4G** | N2 | ~5000 | ~3000 | 5 | 3 |
|  | *npr-1(ad609)* | ~8000 | ~3000 | 8 | 3 |
|  | *gcy-35;npr-1(ad609)* | ~5000 | ~6000 | 5 | 6 |
| **S4B** | N2 | 177 | 133 | 5 | 4 |
|  | *npr-1(ad609)* | 167 | 79 | 5 | 4 |
|  | *gcy-35;npr-1(ad609)* | 182 | 100 | 5 | 4 |

**Table S11: Number of animals and experimental data**
