# Supplementary material for: Synergism between soluble guanylate cyclase signaling and neuropeptides extends lifespan in the nematode Caenorhabditis elegans
Source: Aging Cell. 2017 Jan 4;16(2):401–13. doi: 10.1111/acel.12569 (PMC5334569; doi:10.1111/acel.12569)
Supplement: Supplementary file 16 — Appendix S1 Supplemental Experimental Procedures. [file ACEL-16-401-s016.docx]

**Supplemental Experimental Procedures**

**Strains**

These include the following

| EVG | Genotype | Parental strain /remarks |
| --- | --- | --- |
| EVG037 | N2 |  |
| EVG034 | *npr-1 (ad609) X* | AX204 |
| EVG351 | *gcy-35(ok769) I* | AX1295 |
| EVG281 | *gcy-35(ok769) I;npr-1(ad609) X* | AX1295 |
| EVG350 | *gcy-33(ok323) V* | AX326 |
| EVG186 | *gcy-33(ok323) V;npr-1(ad609) X* | AX326 |
| EVG364 | *gcy-35(ok769) I;gcy-33(ok323) V;npr-1(ad609) X* | AX326 |
| EVG677 | *npr-1 (ad609) X gcy-36 (ok2208)* | RB1729 |
| EVG043 | *hif-1(ia4) V* | ZG31 |
| EVG382 | *hif-1(ia4)V; npr-1(ad609)X* | ZG31 |
| EVG390 | *gcy-35(ok769) I; hif-1(ia4) V; npr-1(ad609) X* | ZG31 |
| EVG381 | *tax-4(ks28) III* | FK103 |
| EVG395 | *tax-4(ks28) III; npr-1(ad609) X* | FK103 |
| EVG396 | *gcy-35(ok769) I;tax-4(ks28) III; npr-1(ad609) X* | FK103 |
| EVG366 | *gcy-35(ok769) I;npr-1(ad609) X;Ex[Pgcy37::gcy-35cDNA::polycismCherry;PF15E11.1::GFP]* |  |
| EVG415 | *npr-1(ad609) X;Ex[Pnpr-1::npr-1::polycismCherry;PF15E11.1::GFP]* |  |
| EVG420 | *gcy-35(ok769) I; npr-1(ad609) X; Ex[Pnpr-1::npr-1::polycismCherry;PF15E11.1::GFP]* | EVG415 |
| EVG487 | *npr-1(ad609) X;Ex[Pgcy-37::npr-1::polycismCherry;PF15E11.1::GFP]* |  |
| EVG520 | *gcy-35(ok769) I; npr-1(ad609) X;Ex[Pgcy-37::npr-1::polycismCherry;PF15E11.1::GFP]* |  |
| EVG442 | *unc-64(e246) III; dbEx[Pgcy-37::YC2.60+ccRFP]* | AX3453 |
| EVG291 | *unc-64(e246) III; npr-1(ad609) X; dbEx[Pgcy-37::YC2.60+ccRFP]* | AX3453 |
| EVG443 | *gcy-35(ok769) I; unc-64(e246) III;npr-1(ad609) X; dbEx[Pgcy-37::YC2.60+ccRFP]* | AX3453 |
| EVG359 | *daf-2(el370) III* | CB1370 |
| EVG532 | *daf-2(el370) III; npr-1 (ad609) X* | CB1370 |
| EVG573 | *gcy-35(ok769) I; daf-2(el370) III; npr-1 (ad609) X* | CB1370 |
| EVG360 | *daf-16(mu86) I* | CF1038 |
| EVG534 | *daf-16(mu86) I; npr-1(ad609) X* | CF1038 |
| EVG468 | *daf-16(mu86) gcy-35(ok769) I; npr-1(ad609) X* | CF1038 |
| EVG470 | *egl-9(sa307) V* | JT307 |
| EVG530 | *egl-9(sa307) V, npr-1(ad609) X* | JT307 |
| EVG572 | *gcy-35(ok769) I; egl-9(sa307) V, npr-1(ad609) X* | JT307 |
| EVG613 | *gcy-35(ok769) I; npr-1 (ad609) x; Ex [RNAi Pflp-17::cDNA gcy-33+CCRFP]* |  |
| EVG715 | *Ex [RNAi Pflp-17::cDNA gcy-33+CCRFP]* | EVG613 |
| EVG721 | *npr-1 (ad609) x; Ex [RNAi Pflp-17::cDNA gcy-33+CCRFP]* | EVG613 |
| EVG608 | *gcy-35(ok769) I; npr-1 (ad609) x; Ex [RNAi Pgcy-37::cDNA gcy-33+PF15E11.1::GFP]* |  |
| EVG713 | *Ex [RNAi Pgcy-37::cDNA gcy-33+PF15E11.1::GFP]* | EVG608 |
| EVG714 | *npr-1 (ad609) x Ex [RNAi Pgcy-37::cDNA gcy-33+PF15E11.1::GFP]* | EVG608 |
| EVG584 | *kyIs536[flp-17::p17 SL2 GFP, elt-2::mCherry]; kyIs538[glb-5::p12 SL2 GFP, elt-2::mCherry]* | CX11697 |
| EVG673 | *npr-1(ad609) x; kyIs536 [flp-17::p17 SL2 GFP, elt-2::mCherry]; kyIs538[glb-5::p12 SL2 GFP, elt-2::mCherry]* | CX11697 |
| EVG679 | *gcy-35(ok769) I; npr-1(ad609) x; kyIs536[flp-17::p17 SL2 GFP, elt-2::mCherry]; kyIs538[glb-5::p12 SL2 GFP, elt-2::mCherry]* | CX11697 |
| EVG005 | *lin-15B(n765) qaIs2241X* | CX7102 |
| EVG636 | *npr-1(ad609) lin-15B(n765) qaIs2241X* | CX7102 |
| EVG685 | *gcy-35(ok769) I; npr-1(ad609) lin-15B(n765) qaIs2241X* | CX7102 |
| EVG688 | *unc-13(e450) I* | CB450 |
| EVG689 | *unc-13(e450) I;npr-1(ad609) X* | CB450 |
| EVG702 | *gcy-35(ok769) unc-13(e450) I;npr-1(ad609) X* | CB450 |
| EVG701 | *unc-31(e928) IV* | DA509 |
| EVG710 | *unc-31(e928) IV;npr-1(ad609) X* | DA509 |
| EVG712 | *gcy-35(ok769) I;unc-31(e928) IV;npr-1(ad609) X* | DA509 |
| EVG891 | *gcy-35(ok769)I; npr-1(ad609)X; Ex[Pgcy-34::npr-1::polycismCherry;Pf15e11.1::GFP]* |  |
| EVG892 | *gcy-35(ok769)I; npr-1(ad609)X; Ex[Pgcy-34::gcy-35(cDNA)::polycismCherry;Pf15e11.1::GFP];* |  |
| EVG895 | *gcy-35(ok769)I; npr-1(ad609)X; Ex[Pglr-3::egl-1::polycismCherry;Pf15e11.1::GFP]* |  |
| EVG939 | *Ex[Pglr-3::egl-1::polycismCherry;Pf15e11.1::GFP]* | EVG895 |
| EVG933 | *npr-1(ad609)X; Ex[Pglr-3::egl-1::polycismCherry;Pf15e11.1::GFP]* | EVG895 |
| EVG897 | *gcy-35(ok769)I; npr-1(ad609)X; [RNAi Pgcy-34::cDNA gcy-33+PF15E11.1::GFP]* | EVG608 |
| EVG917 | *qqIR1(X,CB4856>N2) [npr-1(215F)]* | QX1155 |
| EVG918 | *gcy-35(ok769)I;qqIR1(X,CB4856>N2) [npr-1(215F)]* | QX1155 |
| EVG934 | *gcy-35(ok769)I; egl-3(n150) V; npr-1(ad609)X)* | MT150 |

In general, all of the strains we used in this manuscript were either generated from our own laboratory N2 strain or outcrossed with it at least twice.

**Lifespan analysis**

To generate synchronized worms, we collected eggs from gravid hermaphrodites using hypochlorite solution ([Brenner 1974](#_ENREF_4)). The collected embryos were put in M9 buffer and rotated at 21°C for ~16 h. The hatched L1 larvae were grown on NGM plates containing OP50 bacteria until the young adult stage (we defined the L4 moult as t = 0 in our lifespan data analysis, as described in ([Apfeld & Kenyon 1999](#_ENREF_3))). For each biological repeat in a lifespan experiment, we transferred at least 40 young adults into five experimental plates (eight worms per plate). The experimental plates were seeded with 50 μL of fresh OP50 bacteria (grown in 2XTY medium to an OD600~0.7) 2-7 days before the experiment unless otherwise mentioned. For studies with UV-killed bacteria, the freshly seeded plates were exposed to UV radiation of 0.9999 joules/cm^2^ for 10 min using Hoefer™ UVC 500 Ultraviolet Crosslinker (Hoefer Pharmacia, Inc.). For lifespan assays in the presence of FUDR assays/live bacteria, the NGM plates were supplemented with 50 µM FUDR and seeded with 50 μL of fresh OP50 bacteria. In these experiments, the L1 larvae were grown on regular NGM plates, and transferred to the NGM-FUDR plates at the L4 stage. For lifespan assays in the presence of FUDR assays/killed bacteria, we supplemented the NGM plates with 50 µM FUDR and 100 µg/μL ampicillin (Amp). These plated were seeded with 10 fold concentrated OP50, and exposed to UV radiation (as described above). In these experiments, the L1 larvae were grown on NGM plates containing UV-killed bacteria and transferred to FUDR/Amp/UV-killed bacteria at the L4 stage. For experiments with butylated hydroxyanisole (BHA), we adopted the protocol from Temmerman and colleagues ([De Haes *et al.* 2014](#_ENREF_6)). In brief, the NGM plates were supplemented with 25 μM BHA, and the worms were grown on these plates one generation before the experiment. Since the BHA plates also contained 0.1% ethanol (EtOH), the control plates for these experiments contained 0.1% EtOH. Notably, animals in the control experiments were also grown for one generation on NGM+0.1% EtOH plates. For lifespan experiments with 4-hydroxy-2,2,6,6-tetramethylpiperidin-1-oxyl (tempol) and paraquat, the NGM plates were supplemented with either 5 mM tempol or 0.1 mM paraquat. In these experiments, the worms were transferred to the tempol or paraquat plates at day 1 of adulthood. For lifespan experiments at 1%, 11%, and 35% O_2_ we used O_2_/N_2_ gas mixtures (Moshlion Oxygen Jerusalem LTD). Lifespan experiments at 11% and 35% O_2_ were performed in a hypoxia chamber from StemCell Technologies (Inc), and experiments at 1% O_2_ in a hypoxic glove box (Coy Laboratory Products). In the course of these experiments, we briefly exposed the worms to 21% O_2_ during scoring, and transferred to new plates. Worm survival was scored every two days for live, dead (when it no longer responded to touch), and missing worms. To avoid progeny contamination, we transferred the worms to fresh plates every 2 days until the post-fertile stage, and then every 4 days until the end of experiment. Worms that displayed internal progeny hatching (worm bagging), ruptured, burrowed in the agar, or crawled off the plates were censored. However, they were included in the lifespan data analysis as censored animals (as described in ([Lee *et al.* 2010](#_ENREF_10))). All life span studies were performed at 21°C unless otherwise mentioned.

**Molecular biology**

***Transgenes***

In brief, we used modified polycistronic mCherry pPD95.75 expression vector ([Gross *et al.* 2014](#_ENREF_8)) to express *npr-1* and *gcy-35* in specific neurons. Microinjections were performed as described previously ([Mello *et al.* 1991](#_ENREF_11)). We amplified *npr-1* promoter (6.8 Kb) and *npr-1(215V)* allele using N2 genomic DNA. The promoter region was amplified with 5’ GTA CGA ACC TAG GCT AAA ACT CGG GCTTAA ACC TAT CTG AGT A 3’ and 5’ GTG GGT TGG TAC CAG TCA CGT TTA ATT AAT TGG CCT ATG TCT GAA ATT TTT TTA GTC TAA TGG 3’, and inserted using AvrII and KpnI. We introduced an internal PacI restriction site in the reverse primer for *npr-1(215V)* subcloning. The *npr-1(215V)* allele was amplified using 5’ GTA CGA ATT AAT TAA AAA ATA TAA TGG AAG TTG AAA ATT TTA CCG ACT GTC AAG TAT 3’ and 5’ GTG GGT TGG TAC CTC AGA CTA GCG TGT CGT TGA CGC TG 3’ and inserted with PacI and KpnI. To express *npr-1(215V)* in AQR, PQR, and URX, we used *gcy-37* promoter region. The 1.34 kb promoter region was amplified using 5’ GGT GGT TCG ATA TCG GGA CCG ACA TGC TCG GCT TG 3’ and 5’ GTG GGT TCC TAG GCT GAA TTT ACT TTC TTT TTT TTT TGA AAT TTT AGC T 3’, and inserted with EcoRV and AvrII.

Here, *npr-1(215V)* allele was amplified using 5’ GTA CGA ACC TAG GAA AAT ATA ATG GAA GTT GAA AAT TTT ACC GAC TGT CAA GTA T 3’ and 5’ GTG GGT TGT CGA CTC AGA CTA GCG TGT CGT TGA CGC TG 3’ and inserted with AvrII and SalI. Both *npr-1(215V)* expression constructs were injected to *npr-1(ad609)* worms, with PF15E11.1::GFP co-injection marker at 2.5 ng/μL and 47.5 ng/μL, respectively. To restore *gcy-35* function in AQR, PQR, and URX, we replaced the *npr-1(215V)* genomic region (just after *gcy-37* promoter) with *gcy-35* DNA coding sequence. The coding region of *gcy-35* was amplified using 5’ GTA CGA ACC TAG GAA AAT ATA ATG TTC GGC TGGATT CAC GAA AGC TTC 3’ and 5’ GTG GGT TGT CGA CTT AAG AAA TTG TGC AAG TCG GTG ATC GGA TG 3’ and inserted with AvrII and SalI. The *gcy-35* expression construct was injected to *gcy-35;npr-1(ad609)* worms, with the PF15E11.1::GFP co-injection marker at 2.5 ng/μL and 47.5 ng/μL, respectively. To express *npr-1(215V)* in AQR, PQR, and URX, we used also *gcy-34* promoter region. The 0.75 kb promoter region was amplified using 5’ GTA CGA ACC TAG GCT TTC CGT TTT ATC AAC TAT TCA ATC TGT CAT CG 3’ and 5’ GTG GGT TTT AAT TAA TTT GAG AAG TTT TTT GAA CAG CTG CAG AG 3’, and inserted with AvrII and PacI. To express *gcy-35* in AQR, PQR, and URX, we used also *gcy-34* promoter region. The 0.75 kb promoter region was amplified using 5’ GTA CGA AGA TAT CCT TTC CGT TTT ATC AAC TAT TCA ATC TGT CAT CG 3’ and 5’ GTG GGT TCC TAG GTT TGA GAA GTT TTT TGA ACA GCT GCA GAG 3’, and inserted with EcoRV and AvrII. The constructs that contain the *gcy-34* promoter were injected to *gcy-35;npr-1(ad609)* worms, with the PF15E11.1::GFP co-injection marker at 2.5 ng/μL and 47.5 ng/μL, respectively. The *npr-1* and *gcy-35* constructs were verified by sequencing using the primers listed in Table S10.

For genetic ablation of *egl-1* in RIA, we used the *glr-3* promoter region. The 5.1 kb promoter region was amplified using 5’ GTA CGA ATC CGG AGA ATC GAA GGT TGG AGA GAA ATA TAC 3’ and 5’ GTG GGT TCC TAG GAT GTT AAT AGC AAA TAT TGA AGA TTC TAA AC 3’ and inserted with BspEI and AvrII. *egl-1* was amplified using 5’ GTG GGT TCC TAG GAA AAT ATA ATG CTG GTA AGT CTA GAA ATT ATT TAT TTT TG 3’ and 5’ GTA CGA AGG TAC CTT AAA AAG CGA AAA AGT CCA GAA GAC G 3’ and inserted with AvrII and KpnI. The construct was injected to *gcy-35;npr-1(ad609)* worms, with the PF15E11.1::GFP co-injection marker at 2.5 ng/μL and 47.5 ng/μL, respectively. The *egl-1* constructs were verified by sequencing using the primers listed in Table S10.

***Cell specific RNAi by PCR fusion***

To knock down *gcy-33* in the AQR, PQR, and URX neurons and in the BAG neurons we used the method described in Esposito *et al* 2007 ([Esposito *et al.* 2007](#_ENREF_7)). To amplify the coding region of *gcy-33*, we used a plasmid, from the Yuji Kohara’s plasmid collection, containing the coding region of *gcy-33*. The promoter regions of *gcy-37* (1.34 kb) and *flp-17* (3.3 kb) were amplified from N2 genomic DNA. To knock-down *gcy-33* in AQR, PQR, and URX, the sense and antisense RNAi strands were injected to *gcy-35;npr-1(ad609)* worms at 25 ng/μL each, with the PF15E11.1::GFP co-injection marker (20 ng/μL). To knock-down *gcy-33* in BAG, the sense and antisense RNAi strands were injected to *gcy-35;npr-1(ad609)* worms at 50 ng/μL (each) together with 25 ng/μL ccRFP co-injection marker. The primers used for RNAi by PCR fusion are listed in Table S10.

**RNA extraction and quantitative RT-PCR (qPCR)**

We extracted total RNA from ~120 worms on day 1 and 5 of adulthood that were grown on regular NGM plates or plates containing 5mM tempol. We collected the worms with PBS buffer, and washed them three times with the same buffer. Finally, we centrifuged the worms, replaced the PBS with 200μl of TRIzol Reagent (life technologies, Thermo Fisher Scientific Inc. Waltham, MA, USA), and froze the tubes overnight at -80°C. We thawed the tubes on ice, added the lysates into Eppendorf tubes containing Bullet Blender® pink beads (Next Advance, Inc.), and homogenized the worms using Bullet Blender® Homogenizer Cell Disrupter (Next Advance, Inc.). Total RNA was isolated from the lysates using NucleoSpin® RNA purification kit (MACHEREY-NAGEL GmbH & Co. KG). cDNA was synthesized with iScript™ cDNA Synthesis Kit (Bio-Rad Laboratories Ltd). To quantify gene expression, qPCR was performed using the iTaq™ Universal SYBR® Green Supermix (Bio-Rad Laboratories Ltd). We ran the following program on the Bio-Rad CFX ConnectTM Real-Time System: Initial 3 minutes at 95°C followed by 39 cycles of 10 seconds at 95°C and 30 seconds at 60°C, and finally 10 seconds at 95°C. At the end of each qPCR experiment, we analyzed the melt curve (carried out between 60°C to 95°C) to confirm the specificity of amplicons. Gene expression was normalized to *ned-8* and *Y54G2A.1*, and analyzed using Bio-Rad CFX Manager 3.1 software. The primer sequences of the normalizing and experimental genes are shown in Table S10.

**Speed measurements**

We measured worm speed in the presence and absence of food. For ON-food measurements, we put 8-10 young adult worms on 5 cm low-NGM plates (0.13 g/L bacto-peptone) seeded with 20 μl OP50 bacteria (OD600 ∼ 0.6) 2 days before the experiment. Then, 500 μm deep rectangular PDMS (12 mm wide, 0.5 mm deep, 17 mm long) was put on top of the bacterial lawn with the worms in order to trap the animals inside. We used a PHD 2000 syringe pump (Harvard Apparatus) to deliver humidified gases to the microfluidic chambers at a flow rate of 0.5 ml/min. In addition, we used teflon valves, controlled by a ValveBank Controller (Automate Scientific), to rapidly switch between different O_2_/N_2_ gas mixtures. For OFF-food assays, we directly picked 8 worms into 100 μl M9 buffer drop. We replaced the buffer 5 times, during the course of 30 minutes (to completely remove bacteria). Then, we placed the worms at the center of a 1.7-cm-diameter copper ring on an unseeded low-NGM plate, waited 5 minutes and started the experiment. For each strain, we measured at least 48 animals in 6 independent sets (for both ON and OFF food experiments). We recorded the videos using a Q-Imaging MicroPublisher 5.0 RTV Microscope Camera (QImaging, RHos) mounted onto an Olympus SZ61 stereo microscope (Olympus). The videos were taken at 0.5 frames/s. Video analysis was performed using custom-written MATLAB software (Gross et al, 2014).

***P. aeruginosa* (PA14) killing assay.**

*P. aeruginosa* PA14 colonies from freshly streaked LB plate supplemented with 100 μg/ml ampicillin were inoculated into 2XTY medium, and grown to an OD600~0.7. Then, we seeded 50 μl bacteria to each experimental plate, and incubated it at room temperature for 2 days. For each experiment, we transferred 8 worms (on day 1 or 5 of adulthood) to the plate, and scored live, dead (when it no longer responded to touch), and missing worms every 8-16 h. The experiments were performed at 20°C.

**Heat shock assay.**

For each experiment, we put 8 worms (on day 1 or 5 of adulthood) on NGM plate seeded with 50 μL of fresh OP50 bacteria (as described above) and transferred to 35°C. We scored live, dead (when it no longer responded to touch), and missing worms every 8-16 h. The total number of worms was at least 120.

**UV stress assay.**

Non-seeded NGM plates, containing 50 worms (on day 1 or 5 of adulthood), were UV irradiated at 5 mJ/cm^2^ for 10 sec in a Hoefer™ UVC 500 Ultraviolet Crosslinker (Hoefer Pharmacia, Inc.). After irradiation, we transferred the worms to lifespan assay plates (as described above) and scored live, dead (when it no longer responded to touch), and missing worms every 24 h.

**ATP content.**

For each ATP measurement, we collected approximately 1000 worms (on day 1 or 5 of adulthood), washed them 3 times with ice cold M9 buffer, boiled for 15 min (to inactivate ATPases), and immediately froze at -80°C until use. After thawing, we added Bullet Blender® pink beads (Next Advance, Inc), broke the worm cuticle with the Bullet Blender® Homogenizer Cell Disrupter (Next Advance, Inc) and sonicated them in Citizen^©^ ultrasonic cleaner for 8 min. After centrifugation (13,300 xg for 15 min), the clear lysate was used for ATP determination using luminescent ATP detection assay kit (Abcam Inc). The ATP levels were normalized to the protein content of the sample using BCA protein assay kit (Pierce).

**Measurement of developmental timing.**

Plates with many gravid hermaphrodites were washed with M9 buffer five times to remove all the worms. The remaining eggs were allowed to hatch for 1 h. The newly hatched L1 animals were transferred onto new plates. Once they reached L4 stage, 1-3 worms were transferred to each well of a 24-well NGM plate seeded with 25 μL OP50 bacteria per well. Each worm was monitored every hour and scored as adult when it had at least one egg in its body.

The experiments were performed at 21°C. For each strain, we measured at least 38 animals. The measurements were divided to 3 independent sets that were conducted on various days.

**Egg laying assay.**

The egg-laying protocol was adopted from Craig *et al* 2012 ([Craig *et al.* 2012](#_ENREF_5)) with a few modifications. We used 24-well NGM plates seeded with 15 μL OP50 bacteria per well. We transferred 2 worms to each well (24 h-post L4 stage (day 1) or at 120 h-post L4 (day 5)). After 4 h, we removed the worms, and counted the number of eggs laid. These experiments were performed at 21°C.

**Bordering assay.**

Bordering experiments were performed as described previously ([Abergel *et al.* 2016](#_ENREF_1)). In brief, 40 young adult hermaphrodites (grown in 21% O_2_ at room temperature) were transferred to a NGM plate that was seeded 2 days before with 50 μL OP50 bacteria (OD600~0.6). The assay plates were put in 21% O_2_ at R/T for 1 h. Bordering index shows the fraction of worms found on the bacterial lawn border divided by the total number of worms on the plate, multiplied by 100.

**Measurement of protein oxidation**

We quantified protein oxidation using OxyBlot Protein Oxidation Detection Kit (EMD Millipore) according to the manufacturer's instructions, with several modifications. Briefly, approximately 1000 worms (on day 1 or 5 of adulthood) were picked, washed 3-5 times with M9 buffer, which was replaced with 50 μl of lysis buffer (100 mM Hepes pH 7.2, 4% SDS; 25 mM DTT), and frozen at -80°C until use. After thawing, we added Bullet Blender® pink beads (Next Advance, Inc), homogenized the worms using Bullet Blender® Homogenizer Cell Disrupter, and sonicated them for 1 min. We incubated the lysate at 37°C for 30 min, and centrifuged the tubes at 13,300xg for 15 min. We mixed 10 μL from the clear protein lysate with 10 μL DNP solution, and incubated it for 15 min at room temperature. We then neutralized the pH of the samples by adding 7.5 μL neutralization buffer and 4.5 μL 2.5N NaOH. We quantified protein concentration using BCA protein assay kit (Pierce), and separated equal amounts (5-10 μg) on 12% SDS–PAGE gels. For Oxyblot analysis, proteins were transferred to Biotrace^@^ Nitrocellulose membrane (Pall Corporation, Ann Arbor, MI, USA). Prior to the incubation with the anti-DNP antibody, we stained the gel with Ponceau S Solution (0.1% w/v Ponceau S in 1% v/v acetic acid) for 20 min. The densitometry of the Ponceau staining was used as a loading control to normalize the oxyblot density. The Ponceau stain was then completely removed by rinsing the membrane with double-distilled water. Then, the membranes were blocked with 5% skim milk (BD) for 1 h, and incubated with OxyBlot’s rabbit anti-DNP antibody (1:1,000, in 5% skim milk TBST) at 4°C, overnight. After 3 washes with TBST buffer, we added the secondary antibody (1:5,000 HRP-coupled anti-rabbit antibody) for 1 h at room temperature. We used SuperSignal® West Pico Chemiluminescent Substrate (Thermo Fisher Scientific, Inc) to detect signals. Bands intensities were quantified using ImageJ software ([Abramoff 2004](#_ENREF_2)).

**Thrashing assay**

The worms (on day 1 or 5 of adulthood) were put into 96 well plate (single worm per well), containing 50 μl M9 buffer. Thrashes were counted immediately for 30 s (we multiplied the results by 2 to extrapolate thrashing rate per minute). An individual thrash is defined as a complete change in the direction of bending at mid-body.

**Pharyngeal pumping assay**

Individual worms (on day 1 or 5 of adulthood) were picked into NGM plate containing 10 μL bacteria. After 15 min acclimation period, we put a coverslip (18 X 18 X 0.4 mm, Marienfield Laborotory Glassware, Paul Marienfield GmbH and Co. KG) on top of the worms, and measured pharyngeal pumping for 30 s (we multiplied the results by 2 to extrapolate pumping rate per minute).

**Tunicamycin resistance assay**

To induce stress, we picked 50 worms (at day 1 or 5 of adulthood) directly from the NGM plates, washed them 3-5 times with M9 buffer, and put them in 96-well plates. Each well contained 50 μL M9 buffer supplemented with 400 μg/ml tunicamycin (tunicamycin was initially dissolved in DMSO, and then diluted with M9 buffer). Notably, the worms were also treated with M9 buffer+ 0.1% DMSO as a control for tunicamycin exposure. The plates were gently agitated at 21°C, and worm survival was determined after 16 h.

**Oxygen consumption assay**

For O_2_ consumption rate measurements, we picked approximately 300 worms (on day 1 or 5 of adulthood) directly from NGM plates, washed them 3-5 times with M9 buffer, and put them in anaerobic quartz cuvette (Hellma Analytics, Hellma GmbH & Co. KG) containing 250 μL M9 buffer. We modified the cuvette to O_2_ consumption measurements by gluing an O_2_ sensor spot (PreSens Inc. Precision Sensing GmbH, Regensburg, Germany) to the semitransparent side of the cuvette. The changes in O_2_ concentration were monitored by a FIBOX3 single channel fiber optic oxygen transmitter. During the course of the measurement (30 min), the worms were constantly stirred using a magnetic stirrer. The O_2_ consumption rates were normalized to the protein content of the worms (measured by BCA protein assay kit (Pierce)).

**Paraquat resistance assay**

To check worm resistance to paraquat, we washed ~150 worms (on day 1 or 5 of adulthood) from the NGM plates with an M9 buffer, and washed twice more with the same buffer. Then, ~12 worms per well were put in a 96 well plate with 100 µl 200 mM paraquat (in M9) or to M9 as control. The plates were placed on a shaker at 350 rpm at 21°C, and the survival of worms were measured after 1, 3, 6, and 24 h. We performed six independent assays for each strain. The total number of worms for each experiment was at least 120 in 6 independent sets.

**ROS measurement**

To measure ROS, we used the 2’,7’dichlorofluorescin diacetate (DCFDA) dye according to Harding et al, 2003 ([Harding *et al.* 2003](#_ENREF_9)). Briefly, we collected approximately 100 worms (on day 1 or 5 of adulthood), washed them three times with M9 and then resuspended in DCHFDA solution (final 50 µM in M9 buffer). Then the worms were gently agitated for 1 h. The worms were washed three times with M9, immobilized by 25 mM sodium azide and mounted on 2% agarose pads (agarose pre-dissolved in M9 buffer supplemented with sodium azide). The worms were imaged using an Olympus IX71S1F-3-5 inverted microscope equipped with UAPON40X Universal apochromatic water immersion objective (Olympus, Tokyo, Japan). Importantly, to attenuate DCF oxidation by light, we used a combination of neutral density filters (32ND50/32ND12/32ND6). Image analysis was performed using ImageJ (Abramoff et al., 2004).

**References**

Abergel Z, Chatterjee AK, Zuckerman B, Gross E (2016). Regulation of Neuronal Oxygen Responses in C. elegans Is Mediated through Interactions between Globin 5 and the H-NOX Domains of Soluble Guanylate Cyclases. *The Journal of neuroscience : the official journal of the Society for Neuroscience*. **36**, 963-978.

Abramoff MD, Magelhaes, P.J., Ram, S.J (2004). Image Processing with ImageJ. *Biophotonics International*. **11**, 36-42.

Apfeld J, Kenyon C (1999). Regulation of lifespan by sensory perception in Caenorhabditis elegans. *Nature*. **402**, 804-809.

Brenner S (1974). The genetics of Caenorhabditis elegans. *Genetics*. **77**, 71-94.

Craig AL, Moser SC, Bailly AP, Gartner A (2012). Methods for Studying the DNA Damage Response in the Caenorhabdatis elegans Germ Line. *Method Cell Biol*. **107**, 321-352.

De Haes W, Frooninckx L, Van Assche R, Smolders A, Depuydt G, Billen J, Braeckman BP, Schoofs L, Temmerman L (2014). Metformin promotes lifespan through mitohormesis via the peroxiredoxin PRDX-2. *Proceedings of the National Academy of Sciences of the United States of America*. **111**, E2501-2509.

Esposito G, Di Schiavi E, Bergamasco C, Bazzicalupo P (2007). Efficient and cell specific knock-down of gene function in targeted C. elegans neurons. *Gene*. **395**, 170-176.

Gross E, Soltesz Z, Oda S, Zelmanovich V, Abergel Z, de Bono M (2014). GLOBIN-5-dependent O2 responses are regulated by PDL-1/PrBP that targets prenylated soluble guanylate cyclases to dendritic endings. *The Journal of neuroscience : the official journal of the Society for Neuroscience*. **34**, 16726-16738.

Harding HP, Zhang Y, Zeng H, Novoa I, Lu PD, Calfon M, Sadri N, Yun C, Popko B, Paules R, Stojdl DF, Bell JC, Hettmann T, Leiden JM, Ron D (2003). An integrated stress response regulates amino acid metabolism and resistance to oxidative stress. *Molecular cell*. **11**, 619-633.

Lee SJ, Hwang AB, Kenyon C (2010). Inhibition of respiration extends C. elegans life span via reactive oxygen species that increase HIF-1 activity. *Current biology : CB*. **20**, 2131-2136.

Mello CC, Kramer JM, Stinchcomb D, Ambros V (1991). Efficient gene transfer in C.elegans: extrachromosomal maintenance and integration of transforming sequences. *The EMBO journal*. **10**, 3959-3970.
