# Supplementary material for: Synergism between soluble guanylate cyclase signaling and neuropeptides extends lifespan in the nematode Caenorhabditis elegans
Source: Aging Cell. 2017 Jan 4;16(2):401–13. doi: 10.1111/acel.12569 (PMC5334569; doi:10.1111/acel.12569)
Supplement: Supplementary file 17 [file ACEL-16-401-s017.docx]

**Figure S1. The effect of GCY-36 and TAX-4 on N2 and *npr-1(ad609)* worms’ lifespan (related to Figure 1).** (A) Survival curves comparing the lifespans of worm strains. These experiments were performed at 21°C on live OP50. (B) Bright-field and fluorescence images of *gcy-35;npr-1(ad609)* transgenic worms expressing *Pgcy-37::gcy-35(wt)::*polycistronic mCherry (upper panel) or *Pgcy-37::npr-1(215V)::*polycistronic mCherry. The AQR, PQR, and URX neurons are indicated by arrows. Scale bars: 100 μm. (C) Survival curves comparing the lifespan of *gcy-35;npr-1(ad609)* mutants to *gcy-35;npr-1(ad609)* strains expressing either *npr-1(215V)* or *gcy-35(wt)* under the promoter regulation of *gcy-34*. These experiments were performed at 21°C on live OP50. (D-F) Survival curves comparing the lifespans of worm strains at different growth conditions, as indicated by the labels above the graphs.

**Figure S2. GCY-33 is important for *npr-1(ad609)* animals’ extended lifespan (related to Figure 2).** All experiments were performed at 21°C on live OP50. (A) Survival curves comparing the lifespans of worm strains. (B-C) Survival curves comparing the lifespan of N2 and *npr-1(ad609)* worms to N2 and *npr-1(ad609)* transgenic worms expressing *gcy-33* RNAi in AQR, PQR, and URX (under the *gcy-37* promoter) or in BAG (under the *flp-17* promoter). (D-E) Survival curves comparing the lifespan of N2 and *npr-1(ad609)* worms to N2 and *npr-1(ad609)* transgenic worms with ablated AQR, PQR, and URX or BAG. (F) Bordering was measured 60 min after putting the worms on the bacterial lawn. Asterisks indicate significance for comparisons with *npr-1(ad609)* animals. Kruskal–Wallis test with Dunn’s post-test. ****p*<0.001, NS (not significant). Error bars represent SEM.

**Figure S3. The function of NPR-1 in lifespan regulation is modulated by neuropeptide/neurotransmitter signaling (related to Figure 3).** (A-C) Survival curves comparing the lifespans of worm strains at 21°C on live OP50 at 21% O_2_.

**Figure S4. Joint loss-of-function of *npr-1* and *gcy-35* does not increase tolerance to heat, ER UPR, or mitochondrial UPR stress (related to Figure 6).** A comparison between the survival of N2, *npr-1(ad609)*, and *gcy-35;npr-1(ad609)* worms at 35ºC (A), in tunicamycin (B), or in paraquat (C). The number of assays and the number of worms tested in the tunicamycin assay are indicated in Table S3. The statistical information for panels A, and C is shown in Table S1. (D) Representative Oxyblot of protein carbonyls of N2, *npr-1(ad609)* and *gcy-35;npr-1(ad609)* animals at day 1 and 5. The Ponceau staining of the same gel is shown below the Oxyblot. Numbers on the left specify the protein molecular marker size in kDa.
